# Hydrophilic But Not Hydrophobic Surfactant Protein Genetic Variants Are Associated With Severe Acute Respiratory Syncytial Virus Infection in Children

**DOI:** 10.3389/fimmu.2022.922956

**Published:** 2022-07-12

**Authors:** Lynnlee C. Depicolzuane, Catherine M. Roberts, Neal J. Thomas, Keenan Anderson-Fears, Dajiang Liu, João Paulo Pereira Barbosa, Felipe Rodrigues Souza, André Silva Pimentel, Joanna Floros, Chintan K. Gandhi

**Affiliations:** ^1^ Center for Host defense, Inflammation, and Lung Disease (CHILD) Research, Department of Pediatrics, The Pennsylvania State College of Medicine, Hershey, PA, United States; ^2^ Department of Public Health Science, The Pennsylvania State College of Medicine, Hershey, PA, United States; ^3^ Departamento de Química, Pontifícia Universidade Católica do Rio de Janeiro, Rio de Janeiro, Brazil; ^4^ Department of Obstetrics & Gynecology, The Pennsylvania State College of Medicine, Hershey, PA, United States

**Keywords:** respiratory sycytial virus, surfacant protein genetic variant, single nucelotide polymorphisms, SFTPA1, SFTPA2, SFTPB, SFTPC, SFTPD

## Abstract

Respiratory syncytial virus (RSV) is the leading cause of lower respiratory tract infection-related hospitalization in the first year of life. Surfactant dysfunction is central to pathophysiologic mechanisms of various pulmonary diseases including RSV. We hypothesized that RSV severity is associated with single nucleotide polymorphisms (SNPs) of surfactant proteins (SPs). We prospectively enrolled 405 RSV-positive children and divided them into moderate and severe RSV disease. DNA was extracted and genotyped for sixteen specific SP gene SNPs. SP-A1 and A2 haplotypes were assigned. The association of RSV severity with SP gene SNPs was investigated by multivariate logistic regression. A likelihood ratio test was used to test the goodness of fit between two models (one with clinical and demographic data alone and another that included genetic variants). *p* ≤ 0.05 denotes statistical significance. A molecular dynamics simulation was done to determine the impact of the *SFTPA2* rs1965708 on the SP-A behavior under various conditions. Infants with severe disease were more likely to be younger, of lower weight, and exposed to household pets and smoking, as well as having co-infection on admission. A decreased risk of severe RSV was associated with the rs17886395_C of the *SFTPA2* and rs2243639_A of the *SFTPD*, whereas an increased risk was associated with the rs1059047_C of the *SFTPA1*. RSV severity was not associated with SNPs of *SFTPB* and *SFTPC*. An increased risk of severe RSV was associated with the 1A^0^ genotype of *SFTPA2* in its homozygous or heterozygous form with 1A^3^. A molecular dynamic simulation study of SP-A variants that differ in amino acid 223, an important amino acid change (Q223K) between 1A^0^ and 1A^3^, showed no major impact on the behavior of these two variants except for higher thermodynamic stability of the K223 variant. The likelihood ratio test showed that the model with multi-allelic variants along with clinical and demographic data was a better fit to predict RSV severity. In summary, RSV severity was associated with hydrophilic (but not with hydrophobic) SPs gene variants. Collectively, our findings show that SP gene variants may play a key role in RSV infection and have a potential role in prognostication.

## Introduction

Respiratory syncytial virus (RSV) is a major burden to the health of children worldwide. It is the leading cause of lower respiratory tract infection and hospitalization in the first year of life in developed countries (1–5). In the United States, it is the most common viral cause of death in children under 5 years of age. While nearly all children are infected with RSV by 2 years of age, only 2-3% of these need hospitalization and of the hospitalized children, 5-10% require mechanical ventilation (2). In a small subset of infants, the risk of death from RSV is as high as 1% even in developed countries (5). Currently, there is no vaccine available for the prevention of RSV infection. Passive immunization is given using anti-RSV monoclonal antibody prophylactically only to high-risk children; for example, to those, who are born extremely premature (<29 weeks), with certain types of congenital heart diseases, chronic lung disease, and certain immunologic disorders (6).

RSV has a heterogeneous presentation and interactions, among virus, host, and environmental factors, all of which have been implicated in affecting RSV disease severity (7). Though environmental factors, sex, and socioeconomic status play a role in the severity of RSV, the underlying mechanisms of this wide spectrum of contributing factors, have not yet been understood. A study of > 12,000 twins showed that genetic factors contributed approximately 20% in determining RSV severity (8) and multiple genetic studies have shown RSV bronchiolitis–associated loci in genes encoding proteins such as surfactant proteins, toll-like receptors, Vitamin D receptor, and various cytokines (9, 10). Thus, genetic variability may partially explain the individual disease susceptibility to RSV infection.

Pulmonary surfactant, a lipoprotein complex, is essential for normal lung function. It prevents alveolar collapse at low lung volumes by lowering the surface tension at the alveolar air-liquid surface. Its components, and especially the surfactant proteins, play important roles both in surfactant-related functions and in innate immune host defense of the lung. In general, the hydrophobic surfactant proteins (SP-B and SP-C) play important roles in surfactant function and structure, and the hydrophilic surfactant proteins (SP-A and SP-D) in innate immunity, as well as in surfactant function (SP-A) (11–16). Because of the diverse functions of the surfactant proteins, any derangement in their structure, function, and/or composition could lead to the development of a wide variety of pulmonary disorders.

Human SP-A is encoded by two functional genes, *SFTPA1* and *SFTPA2* (16, 17), and several genetic polymorphisms are found frequently in the general population (16, 18–21). SP-B, SP-C, and SP-D are each encoded by a single gene, *SFTPB, SFTPC*, and *SFTPD*, respectively (22), and several polymorphisms have been described for each (23–26). Moreover, SP genetic polymorphisms have been shown to associate with RSV (27–30), as well as other pulmonary diseases, such as neonatal respiratory distress syndrome (RDS) (31–35), cystic fibrosis (CF) (36), chronic obstructive pulmonary disease (COPD) (37, 38), acute respiratory distress syndrome (ARDS) (23), hypersensitivity pneumonitis (39, 40), pediatric acute respiratory failure (ARF) (41), and persistent respiratory morbidity in pediatric ARF survivors (42). However, the majority of previous RSV association studies with SP genetic variants were case-control in design, comparing RSV-infected children with healthy children without RSV infection (27, 28, 30, 43). Predicting which infants are at risk of developing RSV infection is less important because virtually all infants are at risk. Hence, in the current study, we took the unique approach of enrolling only RSV-infected children, regardless of their risk factors, and categorizing them by their disease severity to study associations of severe RSV infection with SP genetic variants.

We hypothesized that severe RSV infection is associated with natural SP genetic variants and such associations, in addition to clinical demographic information, may help to identify at-risk children for severe RSV. We further hypothesized that the genetic variation of two significant SP-A haplotypes has an impact on the molecular dynamics of SP-A protein.

## Materials and Methods

Subjects: We prospectively enrolled and collected blood samples from 416 children (ages between 7 days and 3 years old) admitted with a diagnosis of RSV infection at two academic children’s hospitals, Penn State Health Children’s Hospital and the University of Virginia Children’s Hospital, during three consecutive winters. The diagnosis of RSV was made by either direct fluorescent assay or viral culture of nasopharyngeal swabs. Clinical data were extracted from their medical records, including demographics, risk factors for RSV infection, history of parental smoking, pet exposure, length of stay, and co-infections.

For this study, severe RSV was defined *a priori* as the need for admission to the intensive care unit with or without mechanical ventilation. Children admitted to the general pediatric ward were considered as having moderate RSV infection. In the current study, 171 (42%) and 234 (58%) children were diagnosed with moderate and severe RSV disease, respectively. We observed 32 (out of 171) and 123 (out of 234) cases of co-infection in moderate and severe RSV groups, respectively. Out of the 32 cases of co-infection in the moderate RSV group, 8 cases were of otitis media, 9 were with clinical and radiographic pneumonia of unknown bacterial etiology, 2 cases of each Streptococcus pneumoniae and staphylococcus aureus pneumonia, 3 cases of influenza, 2 cases of sinus infection, 1 case of each adenovirus, Moraxella catarrhalis, group b streptococcus urinary tract infection, Hemophilus influenzae bacteremia, coagulase-negative staphylococcus bacteremia, and clostridium difficile. Out of the 123 cases of co-infection in the severe RSV group, the majority of them were due to bacterial infection, n=112 (Hemophilus influenzae = 31, Moraxella catarrhalis = 27, Streptococcus pneumoniae = 22, Staphylococcus aureus = 16, Klebsiella pneumoniae = 4, Escherichia coli = 3, Bordetella pertussis = 2, Enterobacter cloacae = 2, coagulase-negative staphylococcus =2, Pseudomonas aeruginosa = 1, Neisseria meningitidis = 1, Serratia marcescens = 1); 5 cases were each of viral (influenza = 2, 1 case each of adenovirus, rhinovirus, and parainfluenza) and fungal infections (candida albicans = 5) and 1 case of otitis media. The majority of the co-infections were due to a wide variety of bacteria; hence, we have clumped them together and adjusted them as a co-infection covariate.

For statistical analysis purposes, children with severe and moderate RSV infection were considered as cases and controls, respectively. The protocol to collect human samples in this study was approved by the Human Subjects Protection Office of The Pennsylvania State University College of Medicine and the Institutional Review Board for Health Sciences Research at The University of Virginia, and informed consent was obtained from each parent or guardian.

### DNA Isolation

The samples received from the participating sites, were numbered sequentially upon arrival with no other identifiers. DNA extraction and genotyping, each was performed in a blinded fashion to reduce any potential bias. A total of 416 children were enrolled for the current study, however, eleven subjects were excluded from analysis due to lack of genotyping information secondary to degradation of the original sample quality, leaving the final sample size of 405 subjects.

Genomic DNA was extracted from blood samples using QIAamp Blood kit (Qiagen, Valencia, CA USA) following the manufacturer’s instructions as described earlier (19).

### Selection of Genetic Variants

A total of 16 target SNPs of SP genes, *SFTPA1*, *SFTPA2*, *SFTPB*, *SFTPC*, and *SFTPD* were selected that included, 5 SNPs from *SFTPA1*, rs1059047, rs1136450, rs1136451, rs1059057, and rs4253527; 4 SNPs from *SFTPA2*, rs1059046, rs17886395, rs1965707, and 1965708; 3 SNPs from *SFTPB*, rs2077079, rs3024798, and rs1130866; 2 SNPs from *SFTPC*, rs4715 and rs1124; and 2 SNPs from *SFTPD*, rs721917 and rs2243639. Several acute and chronic pulmonary diseases of all age groups, such as, neonatal RDS (31–35), CF (36), COPD (37, 38), ARDS (23), hypersensitivity pneumonitis (39, 40), pediatric ARF (41), and persistent respiratory morbidity in pediatric ARF survivors (42) have been shown to be associated with the studied SNPs. The SP-A1 (6A, 6A^m^, m=0-13) and SP-A2 (1A, 1A^n^, n=0-15) genotypes were assigned as described (19).

### Genotype Analysis

We used polymerase chain reaction-restriction fragment length polymorphism (PCR-RFLP), as described earlier (19), to analyze *SFTPA1*, *SFTPA2*, *SFTPD* (19, 23), *SFTPB* (23, 36), and *SFTPC* (44) gene polymorphisms. The PCR primer sequences and restriction enzymes used are given in **Supplementary Table 1** and the detailed method is described elsewhere (36). This method was used for genotyping of approximately half of the samples. The other half of the samples were processed using a multiplexed polymerase chain reaction workflow of Ampliseq utilizing custom designed panels from Illumina, (Illumina, San Diego, CA) (**Supplementary Table 2**). The library was prepared according to manufacturer’s instructions. All reagents for the library preparation were from Illumina, San Diego, CA, unless specified otherwise. Briefly, 20 nanograms of DNA (in low EDTA TE buffer solution, 10 mM Tris-HCl +1 mM EDTA, pH 7.0) were used and mixed with 4.5 µl of ampliseq Hi-fi mix (Illumina, San Diego, CA) and 2 µl of custom primer design panels to a final volume of 20 µl (**Supplementary Table 2**). PCR was performed at 99°C for 2 min, 21 cycles of 99°C for 15 seconds, and 60°C for 8 minutes (instead of 4 minutes recommended in manufacturer’s instructions) to optimize amplification of the studied genes. Next, we used 2 µl of the FuPa Reagent (Illumina, San Diego, CA) to digest primer dimers and amplicons. The library prep was vortexed and then centrifuged briefly at 280 x g for 10 seconds. The following volumes of reagents were added in the order listed to each sample (switch solution - 4 µl, unique index adapters for each sample - 2 µl, and DNA ligase – 2 µl) to the 22 µl of amplicons to make the final volume of 30 µl to ligate the index adapters. The ligation program was performed at 22°C for 30 minutes, 68°C for 5 minutes, and then at 72°C for 5 minutes. Libraries were cleaned up using AMPure XP beads per manufacturer’s instructions (Beckman Coulter, CA, USA) and amplified a second time after adding 1X Lib Amp Mix (45 µl) and 10X Library Amp primers (5 µl) at 98°C for 2 min, 7 cycles of 98°C for 15 seconds, 64°C for 1 minute. The second cleanup of libraries was done using AMPure XP beads as noted above and sequenced at the Penn State College of Medicine Genome Sciences Facility (Hershey, PA). SNPs, rs1059047, rs1136450, rs2243639, were not included in the final analysis because it was challenging to make appropriate calls with the sequencing method.

### Statistical Analysis

We used descriptive statistics to define the study population and a t-test to compare the two groups (moderate vs severe). For the genetic analysis, we used a total of 5 dummy variables to represent the six ancestral covariates, i.e. Hispanic, Black, Asian, others (Hawaiian and Pacific Islanders), and Mixed. The sixth ancestral category, White, was used as the baseline (0,0,0,0,0). Using these encodings as well as additional covariates of sex, age, smoking exposure, pet exposure, and co-infection, logistic regression models were constructed for each of the 16 SNPs using PLINK 2.0. Additional models, both logistic regression (PLINK 2.0) and associations (PLINK 1.9), were constructed *via* variation of the covariates as well as including additional interaction and dominant/recessive features.

Given that certain SNPs were in linkage disequilibrium, permutation tests were used to correct for the multiple comparisons. In each permutation, we shuffled the phenotype so that the connections between the phenotype and the genetic variants are broken and a null distribution can be generated. A regression model is then run on each permuted dataset in the same way it is used to analyze the original dataset. The minimal p-value of all tested SNPs in each permuted dataset forms an empirical distribution, which is used to determine the corrected p-value after controlling for the family-wise error rate. The corrected p-value for each SNP is determined by:


corrected P−value=(# of Permuted minimal P−values>Original P−value)/(Total # of Permutatations)

Odds ratios (OR) were calculated for the tested SNPs to determine whether variants were associated with risk (> 1) or protection (< 1). To test the 42 genotypes of multi-allelic variants of SP-A1 and SP-A2 i.e., 1A/1A^0^, 6A/6A^2^, etc, a multivariate logistic model was used including all covariates previously described, following our published approach for analyzing multi-allelic variants in genetic association studies (45). A likelihood ratio test (LRT) was used to compare models of clinical demographic data only and clinical demographic data with genotype information to determine if the multi-allelic variants are associated. Analysis was performed using the R framework, with regression models using both PLINK 2.0 for individual SNP tests and the stats package for multi-allelic models and PLINK 1.9 for individual SNP association tests. The lmtest package was used for likelihood ratio test.

Software Versions:

PLINK – 1.9 & 2.0

R (stats package included) – 4.0.2

Readxl – 1.3.1

Dplyr – 1.0.4

Tidyr – 1.1.2

biomaRt – 2.44.4

forcats – 0.5.1

Biobase – 2.48.0

lmtest – 0.9-48

data.table – 1.14.0

kableExtra – 1.3.4

### Molecular Modeling of Human SP-A

Next, we studied the impact of rs1965708 on the molecular dynamics of SP-A. This SNP changes a glutamine (Q) to lysine (K) at amino acid position 223 of SP-A2. The rationale for studying this particular SNP is based on the following: a) a previous association of RSV with the *SFTPA2* rs1965708 has been observed (43); b) in the current study an association of RSV severity with SP-A2 protein variants was observed, as assessed by the logistic regression analysis (see results below); and c) the location of the rs1965708 SNP is in the SP-A C-terminal carbohydrate recognition domain (CRD), a region shown to bind carbohydrates on the surface of pathogens in a calcium-dependent manner to enable neutralization and clearance of pathogens including RSV (16, 46).

The three-dimensional structure of human SP-A is not available in any database, and one way to solve this problem is to apply comparative/homology modeling. This modeling is based on identifying the three-dimensional structure of known proteins that resemble the structure of the query sequence, producing an alignment between the amino acids of the known structure and the amino acid sequence of the desired structure (47, 48). To obtain the three-dimensional structure of human SP-A, we used as a template, the crystallographic structure of *Rattus norvegicus* SP-A, obtained from the protein data bank (49), code 5FFT (50) with a resolution of 2.20 Å. We obtained the SP-A three-dimensional structure through the Swiss-Model server (48, 51–54), and compared and verified the possible structural errors using the SPDB Viewer (55).

The SP-A variants investigated in the present study consisted of amino acids 104-244 and as such, these variants enabled the study of the amino acid change of interest at residue 223. The variation occurring at the amino acid Q223 was studied by using the PyMOL software by Schrödinger Inc. Applying the mutagenesis tool, we chose the conformation of amino acid K223. The molecular dynamics (MD) simulations of the SP-A K223 and the Q223 variants followed the same protocol and these simulations are described below. One of the smaller SP-A oligomers is a trimer, consisting of three monomer subunits, two SP-A1 and one SP-A2 monomers, i.e., a hetero-oligomer (56) or a homo-trimer consisting of three monomers of either SP-A1 or SP-A2 as shown by electrophoresis under non-denaturing conditions (57, 58). All the gene-specific differences are found in the collagen-like domain of SP-A, specifically at amino acid positions 66, 73, 81, 85 (16, 20, 21). Because the structural information of the first 100 amino acid residues of SP-A is not available in any database, we focused our attention on residues 104-244 to perform MD simulations, as this region contains the amino acid change at position 223 encoded by the rs1965708. An SP-A trimer was created using three identical monomers. The MD simulations mentioned below were performed once with each trimer but we analyzed the data of the three monomers separately in order to study the behavior of each SP-A trimer under various conditions.

MD simulations were performed applying the bonded and non-bonded parameters for the all-atom force field, OPLS-AA (59). The tri-dimensional coordinates and topology of the protein were generated by pdb2gmx, which is part of the GROMACS 2019 package (60–63), and used in this work to perform all MD simulations.

Protein and calcium were placed in an octahedron-shaped box, with a volume of 1393 nm^3^ and filled with 43602 water molecules of the TIP4P model (64–66). Periodic boundary conditions were applied on all axes of the box (67). The system was submitted to the minimization step applying the steepest descent algorithm with the convergence criterion of 100.00 kJ.mol^-1^.nm^-1^ or 20000 steps. The equilibration of pressure and temperature was achieved with a time of 100 picoseconds (ps) following a two-step through the simulation. Towards this we used: (i) the canonical ensemble (NVT) (68), keeping the number of particles, volume, and temperature constant, and (ii) the isothermal-isobaric ensemble (NpT) (69), keeping the number of particles, pressure, and temperature constant. After the equilibration step, the systems were submitted to a production step of 50 nanosecond (ns). All MD simulations were performed at 310 K and 1 bar, using 2 fs of integration time with the lists of pairs being updated at every 5 steps. The cut-off for Lennard Jones and Coulomb interactions was between 0 and 1.2 nm. The leap-frog algorithm was used in the production step with the Nose-Hoover thermostat (70) (*τ* = 0.5 ps) at 310 K and the Parrinello-Rahman barostat (68) (*τ* = 2.0 ps) at 1 bar. All Arg and Lys residues were assigned with positive charges and all Glu and Asp residues were assigned with negative charges. The Visual Molecular Dynamics (71) software were used to visualize the simulation trajectories.

We analyzed the root-mean-square deviation (RMSD), the root-mean-square fluctuation (RMSF), the radius of gyration, hydrogen bond, Dictionary of Secondary Structure of Proteins (DSSP), and principal component analysis (PCA), to assess the conformational influence of the Q223/K223 on SP-A behavior. The RMSD is calculated from the comparison of the overlap of two structures, one of which is taken as a reference (72). For example, in our work, each structure from the molecular dynamic trajectory was compared to the initial structure. The RMSF calculates the fluctuation of each atom of each amino acid from its average position, indicating the flexibility of the amino acids throughout the simulation. The radius of gyration of a protein is the measure of the stability of protein folding. If a protein is stably folded, it will likely maintain a relatively steady value of radius of gyration (Rg). Hydrogen bonds were determined based on the cutoffs of the angle formed between the hydrogen, the donor atom and the acceptor atom and the distance between the donor atom and the acceptor atom. The DSSP computes the secondary structure for each time frame and describes the stability of the secondary structure of proteins throughout the simulation (72). PCA is a statistical technique applied in molecular modeling to reduce the complexity of the data that characterize dominant conformational movements in proteins during molecular dynamics simulations (73). PCA describes them through the significance of the collective movements of the structure that are converted into main movements during the molecular dynamics simulations, that is, the number of dimensions necessary to describe the conformation dynamics is reduced by decomposing its movements from a larger spatial scale to those of a smaller scale (74, 75). For this work, the default values of the gmx hbond module were used from the Gromacs 2019 modules.

The torsional entropy of the proteins was calculated using the gmentro.py program (76). We generated a tabulated data file from the dynamics angle trajectory. Using Gaussian mixtures of the torsional data, gmentro.py was used to calculate the configurational entropy from a conformational ensemble of the whole SP-A structure (76).

## Results

### A. Associations of Severe RSV Risk With SP Polymorphisms and Demographic Variables

**Figure 1:**
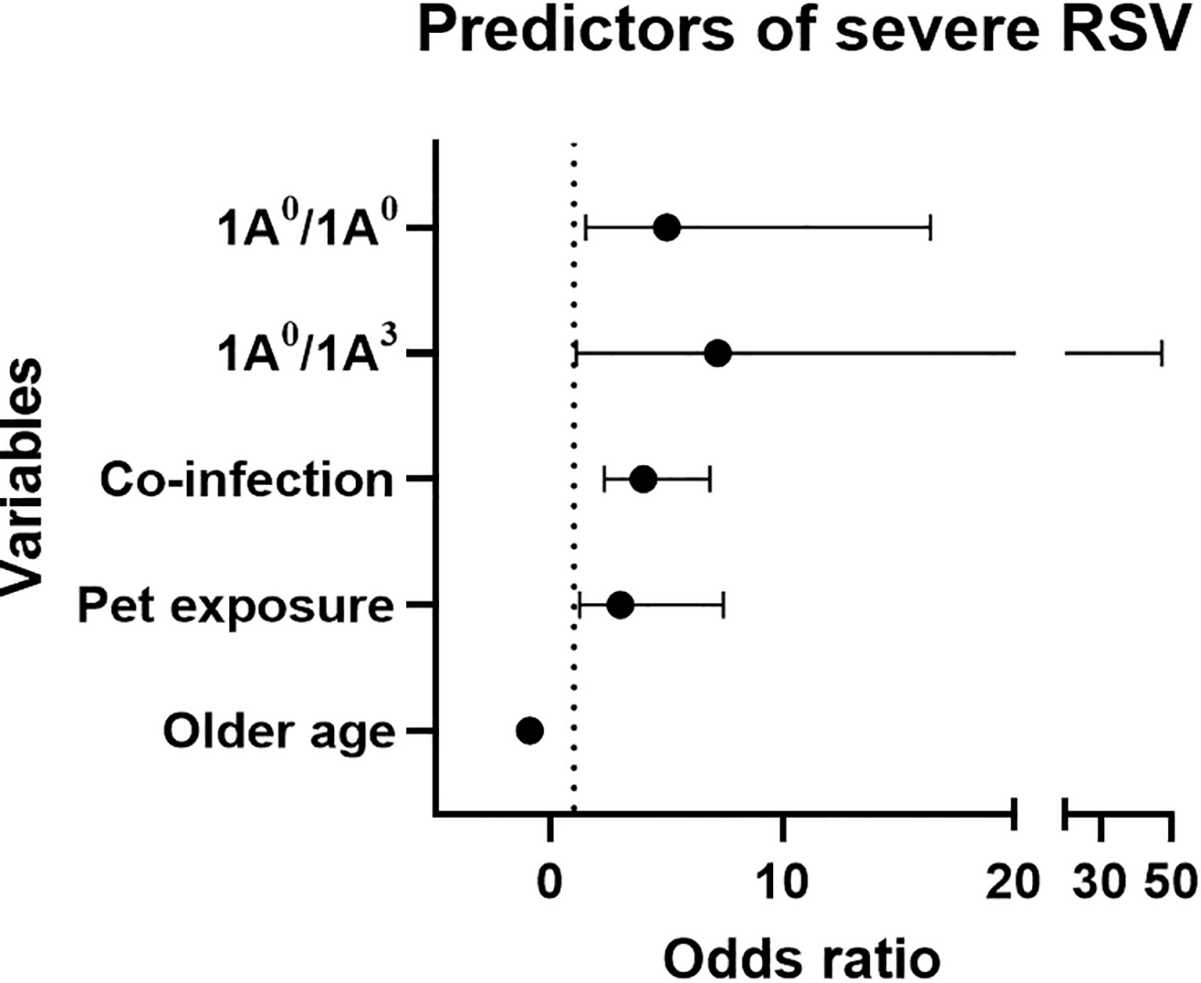
Forest plot represents predictors of severe RSV using mutivariable logistic regression analysis. X-axis and Y-axis represents odds ratio (OR) and variables, respectively. Dotted line represents OR of 1. Based on OR, 1A^0^/1A^0^, 1A^0^/1A^3^, pet exposure and co-infection are associated with increase risk of severe RSV (OR > 1), whereas, older age is associated with decreased risk of severe RSV (OR < 1).

Out of the 405 enrolled RSV-positive children, 171 (42%) and 234 (58%) children were diagnosed with moderate and severe RSV disease, respectively. **Table 1** shows the demographic data describing the study population. There were no statistically significant differences in sex, race, ethnicity, and measures of risk factors previously known to be associated with severe RSV disease (e.g. history of prematurity, congenital heart disease) between the two groups (**Table 1**). The majority of the patients (~ 85%) were white. Infants with severe RSV were more likely to be younger, of lower weight, had exposure to household pets and smoking; as well as more likely to have a co-infection on admission. As one would expect, children with severe RSV required a longer hospital stay and supplemental oxygen at discharge (p < 0.05). The logistic regression analysis of the clinical data showed that history of pet exposure (OR 3.05, 95% CI 1.25-7.46, *p*=0.014), and co-infection on admission (OR 3.98, 95% CI 2.30-6.87, *p* < 0.001) were associated with increased risk of severe RSV, whereas higher age was associated with decreased risk of severe RSV (OR 0.89, 95% CI 0.84-0.94, *p* < 0.001) (**Figure 1**).

**Table 1:** Demographics of the cohort stratified by moderate and severe RSV disease.

Variable	Whole cohort (n = 405)	Moderate RSV (n = 171)	Severe RSV (n = 234)	p value
**Demographics**				
Age on admission (months)	4 ± 5.3	5.35 ± 6.7	3 ± 3.7	<0.001
Weight on admission (kilogram)	5.5 ± 2.4	6.1 ± 2.8	5 ± 2.1	<0.001
Female/male (%/%)	218/187 (54/46)	88/83 (52/48)	130/104 (56/44)	0.45
Non-white race (%)	64 (16)	25 (15)	39 (17)	0.71
Hispanic ethnicity (%)	86 (21)	36 (21)	50 (22)	0.96
**Past history (%)**				
Prematurity	96 (24)	44 (26)	52 (22)	0.43
Cardiac disease	23 (6)	8 (5)	15 (6)	0.62
NICU admission	75 (19)	37 (22)	38 (16)	0.18
Congenital anomalies	29 (7)	16 (9)	13 (6)	0.1
Family history of asthma	166 (41)	77 (45)	89 (38)	0.14
**Environmental exposures**				
Exposure to household smoking	53 (13)	15 (9)	38 (16)	0.03
Pet exposure	53 (13)	8 (5)	45 (19)	<0.001
Co-infection	155 (38)	32 (19)	123 (53)	<0.001
**Hospital days**	8.9 ± 9.9	3.8 ± 2.3	12.7 ± 11.5	0.000
**Duration of support (n=237)**				
Ventilator days	5.7± 5.1		5.7± 5.1	
PICU days	7.8 ± 6		7.8 ± 6	
**Outcomes:** Discharged with				
Supplemental oxygen	24 (6)	5 (3)	19 (8)	0.03
Oral/inhaled corticosteroids	43 (11)	23 (14)	20 (9)	0.08
Bronchodilators	141 (35)	68 (40)	73 (31)	0.08

RSV, Respiratory Syncytial Virus; NICU, Neonatal Intensive Care Unit; Mean ± SD.

The observed frequency distribution of the majority of SNPs shown in **Supplementary Table 3** was in Hardy-Weinberg equilibrium. The logistic regression analysis of SNPs showed that decreased risk of severe RSV associated with two SNPs, the rs17886395_C allele of the *SFTPA2* (OR=0.63, 95% CI 0.46-0.85, *p* = 0.002) and the rs2243639_A allele of the *SFTPD* (OR 0.64, 95% CI 0.41-0.99, *p* = 0.045) after adjusting for significant covariates (age, ethnicity, co-infection, smoke and pet exposure), and increased risk of severe RSV with the rs1059047_C (OR= 3.8, 95% CI 1.1-12.6, *p*=0.032) allele of the *SFTPA1* after adjusting for the same covariates (**Table 2**). As shown in **Supplementary Table 4**, the logistic regression analysis of genotypes after adjusting for the same covariates showed similar association with RSV severity.

**Table 2:** Severe RSV vs moderate RSV using the multivariate logistic analysis after adjusting for covariates (age, ethnicity, co-infection, smoke and pet exposure).

Gene	Allele	OR (95% CI)	*p* value
*SFTPA1*	**rs1059047_C**	**3.80 (1.1-12.6)**	**0.032**
	rs1136450_C	0.68 (0.4-1.3)	0.227
* *	rs1136451_G	0.91 (0.3-2.7)	0.863
* *	rs1059057_G	0.60 (0.1-2.4)	0.472
* *	rs4253527_T	1.40 (0.5-3.9)	0.512
*SFTPA2*	rs1965708_A	0.58 (0.3-1.3)	0.187
* *	rs1965707_T	1.50 (0.7-3.0)	0.273
* *	**rs17886395_C**	**0.63 (0.5-0.8)**	**0.002**
* *	rs1059046_C	1.00 (0.5-2.1)	0.916
*SFTPB*	rs1130866_C	1.00 (0.7-1.5)	0.932
* *	rs3024798_A	0.36 (0.1-1.0)	0.056
* *	rs2077079_C	1.70 (0.6-4.4)	0.313
*SFTPC*	rs4715_A	1.00 (0.5-2.0)	0.959
* *	rs1124_A	0.96 (0.5-1.8)	0.894
*SFTPD*	**rs2243639_A**	**0.64 (0.4-0.9)**	**0.045**
* *	rs721917_C	1.20 (0.8-1.7)	0.427

SNP, single nucleotide polymorphism; OR, Odds ratio; CI, Confidence Interval; Bold text is statistically significant with p ≤ 0.05.

As shown in **Figure 1**, an increased risk of severe RSV was associated with the SP-A2 1A^0^ variant in its homozygous (1A^0^/1A^0^, OR=5, 95% CI 1.5-16.4, *p* = 0.009) or heterozygous form with 1A^3^ (1A^0^/1A^3^, OR=7.2, 95% CI 1.1-47.2, *p* = 0.04).

To evaluate the overall addition of genotype information to demographic data, a likelihood ratio test was done. The model with the genotype information along with demographic data was a better fit to predict disease severity compared to demographic data alone (p = 0.029 and Chi sq of 57.445).

### B. Impact of the *SFTPA2* SNP rs1965708 on SP-A Structure

This SNP encodes the only amino acid change that exists between the SP-A2, 1A^0^ and 1A^3^ protein variants (16, 20, 77). A glutamine (Q) in 1A^0^ is changed into a lysine (K) in 1A^3^ at residue 223, which is located in the CRD of SP-A. Because, in the present study, increased risk of severe RSV was associated with 1A^0^/1A^0^ and 1A^0^/1A^3^ genotypes, we sought to investigate the impact of rs1965708 SNP on SP-A structural parameters *via* the use of SP-A simulated structural data.

**Figure 2:**
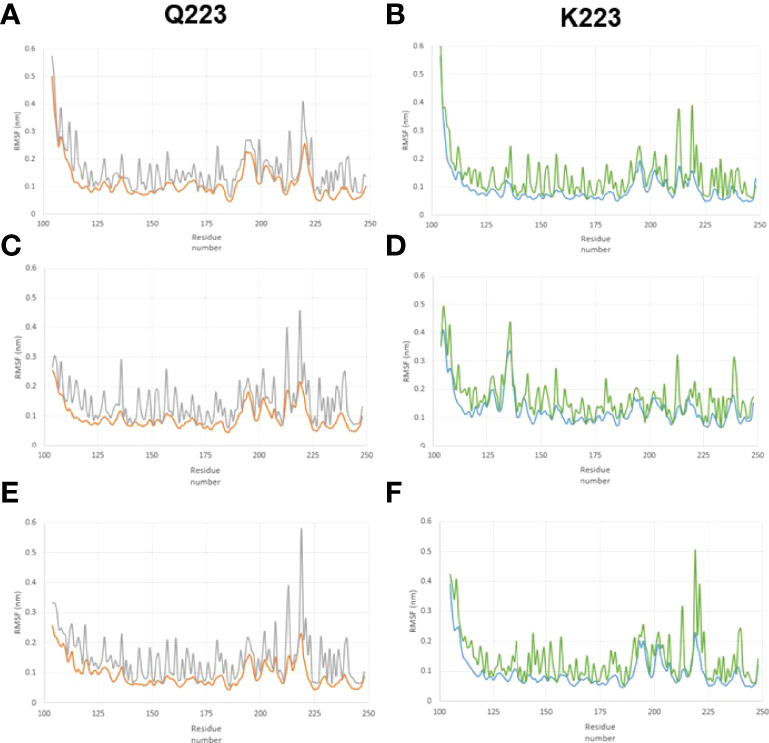
Root-mean-square fluctuation (RMSF) in the backbone and sidechain of the Q223 and K223 SP-A variants. SP-A homo-trimers consisting of three identical monomers of SP-A1 or SP-A2 that included amino acids 104-224 were studied. The RMSF trimer data of each of the two variants (Q223 and K223) were analyzed for each of the 3 identical monomers present in each trimer to study the behavior of SP-A trimer. **(A–C)** represent monomers 1, 2, and 3, respectively, for Q223 trimer, and **(D–F)** represent monomers 1,2,3, respectively, for K223 trimer. The amino acid residue number is on the x-axis and RMSF is on the y-axis. The backbone and sidechain of the Q223 variant, are depicted in orange and gray colors, respectively. Whereas, blue and green colors depict the backbone and sidechain of the K223 SP-A, respectively. The position of peaks did not differ significantly between Q223 and K223 variants, however, the peak of each residue, in general, is greater for Q223 compared to the K223. The region around amino acid 223 (the site of variation), has more intense peaks, indicating a region with great atomic movement.

We studied the RMSF of the Q223 and K223 SP-A variants and **Figure 2** shows the RMSF of the backbone and sidechain of the Q223 and K223 variants. In the RMSF data, although the position of peaks did not differ significantly between Q223 and K223, we observed that the major peaks are in general, greater for Q223 compared to the K223 SP-A variant. The region around amino acid 223 (the site of variation), has more intense peaks indicating a region with great atomic movement. Furthermore, the RMSF values of each of the three identical monomers present in each trimer (Q223 or K223) analyzed did not differ significantly. The sidechain of both Q223 and K223 SP-A monomers showed higher RMSF values than the backbone (**Figure 2**) indicating a greater fluctuation of the sidechain atoms compared to the protein backbone.

The thermodynamic data on the torsional entropy show a larger entropy value for the K223 compared to Q223 SP-A variant (**Table 3**). Although entropy change in the variant process (ΔS_mutation_) seems small (< 70 J K-1 mol-1), it is not negligible and indicates larger stability of the K223 compared to the Q223 SP-A variant. These observations may also be associated with a greater fluctuation mostly in amino acids of the backbone and sidechain atoms of the K223 compared to the Q223 variant.

**Table 3:** First order entropy change (ΔS_mutation_) in the mutation process for protein and for the backbone chain.

	First order entropy (J K^-1^ mol^-1^)	
	Protein	Backbone
**Q223 SP-A**	28331.24	28331.82
**K223 SP-A**	28400.87	28398.73
**ΔS_mutation_**	69.63	66.91

We did not observe a major difference in the RMSD of the center of mass in either Q223 or K223 SP-A (**Supplementary File 1**), indicating that the amino acid change at the specific site (223) at the protein chain does not significantly influence atomic movement in relation to the center of mass. Furthermore, there were no major differences, among the three monomers of each trimer (Q223 and K223), in the radius of gyration (a measure of protein folding stability), the DSSP (a description of secondary structure stability), hydrogen bonds (a measure of overall protein stability), and PCA (to reduce the complexity of the data that characterize dominant conformational movements of the protein during MD simulations). (**Supplementary File 1**).

## Discussion

Risk for RSV has been associated, in several studies, with genetic variants including SP genetic variants. However, most of the previous RSV association studies have used healthy children without RSV infection as controls. In the current study, we took the unique approach of enrolling only RSV-positive children to study association of disease severity with surfactant protein genetic variants. Our findings indicated that 1) a decreased risk of severe RSV is associated with the rs17886395_C of the *SFTPA2* and the rs2243639_A of the *SFTPD*, whereas, an increased risk is associated with the rs1059047_C of the *SFTPA1*. 2) RSV severity is not associated with the studied SNPs of *SFTPB* and *SFTPC*. 3) An increased risk of severe RSV is associated with the 1A^0^ haplotype of *SFTPA2* in its homozygous or heterozygous form with 1A^3^. 4) The model with the SP genetic information along with demographic data is a better fit to predict RSV disease severity compared to demographic data alone. 5) Based on the molecular dynamic studies, the K223 SP-A variant is more stable than the Q223 SP-A (the 1A^3^ haplotype has a lysine (K) and the 1A^0^ has a glutamine (Q) at residue 223).

A decreased risk of severe RSV is associated with the rs17886395_C allele of *SFTPA2* (*p* < 0.002). This SNP results in an amino acid substitution from alanine to proline at position 91 within the coding region (16, 20, 77). Similar to our findings, a decreased risk of RSV in Finnish children (43) and a decreased risk of RDS in white American neonates (35) was associated with the same SNP. Although direct comparison between studies is difficult, due to differences in study design, it is interesting that the rs17886395_C allele is associated with decreased risk of RSV and RDS in different populations and disease processes. It is shown that replacing alanine with proline stabilizes collagen triple helices due to conformational restrictions of the pyrrolidine ring (78). Whether this affects the concentration of a well-functioning SP-A and confers protection against severe RSV, is unknown as direct experimental evidence is lacking.

An increased risk of severe RSV was associated with the rs1059047_C of the *SFTPA1* (*p*=0.032) in our study. This SNP results in an amino acid substitution from valine to alanine at amino acid position 19, also within the coding region (16, 20, 26, 77), although it may or may not be part of all the molecules of the mature SP-A (57). Contrary to our observation, the aforementioned Finnish study found a protective effect of rs1059047_C on RSV disease (43). The difference in findings between the two studies could be due to differences a) in sample size (n=86 compared to n=405 in our study), b) definition of cases and controls, and c) patient population. The Finnish study used RSV-positive children as cases and healthy children without RSV as controls. In contrast, we enrolled only RSV-positive children and divided them between moderate (controls) and severe (cases) RSV disease. The fact that all children get infected with RSV by two years of age, it becomes less important to know who is at risk of getting RSV infection because virtually all children are at risk. For the current study, we enrolled only RSV-positive children to study RSV associations with SP SNPs, and determine who of the RSV-infected subjects is at risk for severe disease. If our findings are replicated and validated independently, the at-risk children for severe RSV could receive disease-modifying treatments without delay and be considered to receive prophylactic anti-RSV monoclonal antibodies in the future. The antibody treatment bears a considerable cost (79) and thus targeting it to those at the highest risk makes economic sense.

A decreased risk of severe RSV was associated with the rs2243639_A allele of *SFTPD* (*p*=0.045). This SNP results in an amino acid substitution from threonine to alanine at position 160 (26). Similar to our findings, previous studies have shown a decreased risk of severe RSV to associate with the rs2243639_A in the Chilean population (30) and in white American children (28). Although the exact mechanisms of how this SNP confers protection against severe RSV is not known, the rs2243639_A is shown to associate with a decreased level of serum SP-D and decreased risk of RDS in premature infants (80). Increased serum SP-D concentration is associated with worst respiratory outcomes in other viral infections, such as influenza and COVID-19 (81, 82), presumably, due to alveolar injury secondary to oxidative stress and infection. We did not measure SPs concentration in our patient population, and hence, we are unable to determine in the present study the impact of the rs2243639, if any, on SP-D concentration and its association with RSV.

An increased risk of severe RSV was associated with the 1A^0^ haplotype of *SFTPA2*, in its homozygous (1A^0^/1A^0^) or in its heterozygous (1A^0^/1A^3^) form. Previous studies have shown varying associations of *SFTPA2* haplotypes with RSV (28, 30, 43). In line with our observation, one study found an association of increased risk of RSV with 1A^0^/1A^3^, but not with 1A^0^/1A^0^ (43). However, another study found an association of decreased risk of severe RSV with the 1A^0^/1A^0^ (29). Although the latter used the same definition for cases and controls as ours, the study population was very different. In that study, approximately 65% of the enrolled patients were African American compared to 85% of Caucasian children in our study. Liu et al. showed differences in frequencies of SP-A haplotypes based on race (83), therefore, the contrasting findings between the two studies could be due to baseline genetic differences in patient population. Furthermore, the sample size (29) was lower (n=277) than that in our study (n=405) and the authors compared the 1A^0^/1A^0^ genotype vs other SP-A genotypes combined due to the low frequency of the other haplotypes in their patient population (29). Nevertheless, the majority of studies have found associations of *SFTPA2* haplotypes with RSV (28–30, 43). Similarly, a previous association study of *SFTPA* genetic variants with severity of Influenza A virus showed that the 1A^0^ of the *SFTPA2* was associated with a need for mechanical ventilation, acute respiratory failure, and acute respiratory distress syndrome in an adult population (84). These findings are not surprising because SP-A2 (encoded by *SFTPA2)* compared to SP-A1 (encoded by *SFTPA1)* exhibits higher activity in innate host defense/inflammatory processes (16).

In addition to RSV infection, the same SNPs and haplotypes have been studied with other infectious diseases such as tuberculosis (85, 86), pneumonia (87), otitis media (88, 89), and allergic bronchopulmonary aspergillosis (26, 90). Of interest, none of the individual studied SNPs were associated with the above-mentioned infectious diseases after correcting for significant covariates. However, similar to our findings, an increased risk of tuberculosis was associated with the 1A^3^ haplotype in Ethiopian (86) and Mexican (85) adult populations. In the above-mentioned studies, there were other haplotypes of SP-A1 and SP-A2 (but not of the 1A^0^ and the 1A^3^ that are observed in the current study) associated with pneumonia, otitis media, and allergic bronchopulmonary aspergillosis. Nonetheless, associations of *SFTPA1* and *SFTPA2* SNPs and haplotypes with infectious diseases other than RSV underline the role of SP-A1 and SP-A2 in innate immunity and host defense functions.

The only amino acid difference, between SP-A2, 1A^0^ and 1A^3^ variants, is generated by the rs1965708 SNP at residue 223, where the amino acid is either a glutamine (Q) in 1A^0^ or a lysine (K) in 1A^3^ (16, 20). Glutamine is a neutral amino acid with a polar amide group, whereas lysine is a positively charged amino acid with a basic side chain. In simulation studies of molecular dynamics, we did not observe any major difference in structure or behavior of the SP-A variants, Q223 and K223, except a higher stability of the K223 as assessed by its higher conformational entropy compared to Q223 SP-A variant. In line with our findings, no significant differences were observed between the Q223K variants in a number of parameters studied *in vitro*, including their ability to form stable secreted complexes, oligomers, structural stability following limited proteolysis, and other (91). Yet, a transgenic mouse model of asthma carrying this SNP showed delayed resolution of eosinophils in bronchoalveolar lavage fluid of SP-A2 223K/K and SP-A knockout mice compared to SP-A2 223Q/Q > wild-type mice (92). It is well known that severe RSV infection early in life can predispose children to a later development of asthma (93, 94). Taken together, we speculate that the 1A^0^/1A^3^ genotype predisposes children to severe RSV secondary to decrease binding and clearance of RSV and may predispose them to develop asthma later in life. In the future, we plan to study the impact of Q223K on the binding ability of SP-A2 to RSV and its subsequent clearance.

Most importantly, a likelihood ratio test revealed that the model that included information about the patients’ genetic variants, along with clinical and demographic data is more accurate at predicting severe RSV infection compared to the model with only clinical and demographic data. The collective observations in this study contribute to an important foundation where, in the future, physicians could consider using this genetic information in clinical decision-making for high-risk children and identify those that may benefit from preventative measures such as an anti-RSV monoclonal antibody or aggressive early treatment such as surfactant replacement therapy.

Strengths of this study include the prospective study design and the well-characterized demographic, illness, and environmental exposure information for the cohort. There are a few limitations of our study. First, the relatively moderate sample size and homogeneous patient population may prevent the generalizability of our findings. Second, we did not serotype RSV in our patient population and RSV genetic variations may have accounted for altered disease severity rather than the host genetic variants (95, 96). Third, we used a direct fluorescent assay and viral culture to diagnose RSV infection instead of molecular testing. Although this holds the possibility of introducing a few false-positive results, a previous study has shown a good correlation between the direct fluorescent assay and molecular approaches, particularly for infants and young children (97). Subclinical infections with RSV are not common. Importantly, in the present study we have only enrolled children who were symptomatic and required hospitalization, hence, RSV was likely causally related to the illness. Therefore, we believe that the method of RSV detection may not change our conclusion. Fourth, we did not measure the level of inflammatory markers or SPs in our patient population, hence, the impact of these SNPs on these markers is unknown. Nonetheless, it is important that the associations observed in the present study be strengthened and validated by increasing the sample size and replicating the findings in other groups of heterogeneous patient populations with RSV, where measurements of other endpoints are performed.

In summary, multiple single SNPs and haplotypes of *SFTPA1, SFTPA2*, and *SFTPD* genes, encoding the hydrophilic SPs, but no SNPs or haplotypes of those (*SFTPB* and *SFTPC*) encoding the hydrophobic SPs were associated with RSV severity, indicating that SPs involved in innate immunity and host defense, play an important role in RSV severity. The model of genetic variants combined with demographic data was a better fit for predicting RSV severity than that of demographic data alone. This observation strengthens the argument that genetic variants do play a role along with environmental factors in the prediction of viral illness severity in children.

## Data Availability Statement

The data presented in the study have been deposited in the ClinVar repository, accession number SCV002506610.

## Ethics Statement

The studies involving human participants were reviewed and approved by Human Subjects Protection Office of The Pennsylvania State University College of Medicine and the Institutional Review Board for Health Sciences Research at The University of Virginia. Written informed consent to participate in this study was provided by the participants’ legal guardian/next of kin.

## Author Contributions

Data curation: CG, LD, CR, NT. Molecular modeling data: JB, FS, AP. Statistical Analysis: KA-F, DL. Methodology: CG, NT, JF. Resources: JF, CG, NT. Supervision: NT, JF. Writing original draft: CG, LD, KA-F, AP. Writing, reviewing & editing: CG, NT, JF. All authors have read and agreed to the published version of the manuscript.

## Funding

The study was supported in part by grants from Children’s Miracle Network to CG and by NIH R37 HL 34788 to JF. The computational work was supported by the Fundação de Amparo à Pesquisa do Estado do Rio de Janeiro (FAPERJ) with the Rio Network of Innovation in nanosystems for the health (Nanohealth) - E-26/010.000983/2019. The National Council for Scientific and Technological Development (CNPq – 465259/2014-6), the Coordination for the Improvement of Higher Education Personnel (CAPES), the National Institute of Science and Technology Complex Fluids (INCT-FCx), and the São Paulo Research Foundation (FAPESP – 2014/50983-3) to ASP.

## Conflict of Interest

The authors declare that the research conducted was in the absence of any commercial or financial relationships construed as a potential conflict of interest.

## Publisher’s Note

All claims expressed in this article are solely those of the authors and do not necessarily represent those of their affiliated organizations, or those of the publisher, the editors and the reviewers. Any product that may be evaluated in this article, or claim that may be made by its manufacturer, is not guaranteed or endorsed by the publisher.

## References

[1] Purcell K Fergie J. Driscoll Children's Hospital Respiratory Syncytial Virus Database: Risk Factors, Treatment and Hospital Course in 3308 Infants and Young Children 1991 to 2002. Pediatr Infect Dis J (2004) 23(5):418–23. doi: 10.1097/01.inf.0000126273.27123.33 15131464

[2] Hall CB Weinberg GA Iwane MK Blumkin AK Edwards KM Staat MA. The Burden of Respiratory Syncytial Virus Infection in Young Children. N Engl J Med (2009) 360(6):588–98. doi: 10.1056/NEJMoa0804877 PMC4829966 19196675

[3] Langley GF Anderson LJ. Epidemiology and Prevention of Respiratory Syncytial Virus Infections Among Infants and Young Children. Pediatr Infect Dis J (2011) 30(6):510–7. doi: 10.1097/INF.0b013e3182184ae7 21487331

[4] Shi T McAllister DA O'Brien KL Simoes EAF Madhi SA Gessner BD. Global, Regional, and National Disease Burden Estimates of Acute Lower Respiratory Infections Due to Respiratory Syncytial Virus in Young Children in 2015: A Systematic Review and Modelling Study. Lancet (2017) 390(10098):946–58. doi: 10.1016/S0140-6736(17)30938-8 PMC5592248 28689664

[5] Stein RT Bont LJ Zar H Polack FP Park C Claxton A. Respiratory Syncytial Virus Hospitalization and Mortality: Systematic Review and Meta-Analysis. Pediatr Pulmonol (2017) 52(4):556–69. doi: 10.1002/ppul.23570 PMC5396299 27740723

[6] Diseases, A.A.o.P.C.o.I Committee, A.A.o.P.B.G. Updated Guidance for Palivizumab Prophylaxis Among Infants and Young Children at Increased Risk of Hospitalization for Respiratory Syncytial Virus Infection. Pediatrics (2014) 134(2):415–20. doi: 10.1542/peds.2014-1665 25070315

[7] Sonawane AR Tian L Chu CY Qiu X Wang L Holden-Wiltse J. Microbiome-Transcriptome Interactions Related to Severity of Respiratory Syncytial Virus Infection. Sci Rep (2019) 9(1):13824. doi: 10.1038/s41598-019-50217-w 31554845 PMC6761288

[8] Thomsen SF Stensballe LG Skytthe A Kyvik KO Backer V Bisgaard H. Increased Concordance of Severe Respiratory Syncytial Virus Infection in Identical Twins. Pediatrics (2008) 121(3):493–6. doi: 10.1542/peds.2007-1889 18310197

[9] Janssen R Bont L Siezen CL Hodemaekers HM Ermers MJ Doornbos G. Genetic Susceptibility to Respiratory Syncytial Virus Bronchiolitis is Predominantly Associated With Innate Immune Genes. J Infect Dis (2007) 196(6):826–34. doi: 10.1086/520886 17703412

[10] Forton JT Rowlands K Rockett K Hanchard N Herbert M Kwiatkowski DP. Genetic Association Study for RSV Bronchiolitis in Infancy at the 5q31 Cytokine Cluster. Thorax (2009) 64(4):345–52. doi: 10.1136/thx.2008.102111 PMC3015100 19131452

[11] Wright JR. Immunoregulatory Functions of Surfactant Proteins. Nat Rev Immunol (2005) 5(1):58–68. doi: 10.1038/nri1528 15630429

[12] Kishore U Greenhough TJ Waters P Shrive AK Ghai R Kamran MF. Surfactant Proteins SP-A and SP-D: Structure, Function and Receptors. Mol Immunol (2006) 43(9):1293–315. doi: 10.1016/j.molimm.2005.08.004 16213021

[13] Serrano AG Pérez-Gil J. Protein-Lipid Interactions and Surface Activity in the Pulmonary Surfactant System. Chem Phys Lipids (2006) 141(1-2):105–18. doi: 10.1016/j.chemphyslip.2006.02.017 16600200

[14] Lopez-Rodriguez E Pascual A Arroyo R Floros J Perez-Gil J. Human Pulmonary Surfactant Protein SP-A1 Provides Maximal Efficiency of Lung Interfacial Films. Biophys J (2016) 111(3):524–36. doi: 10.1016/j.bpj.2016.06.025 PMC4982931 27508436

[15] Depicolzuane L Phelps DS Floros J. Surfactant Protein-A Function: Knowledge Gained From SP-A Knockout Mice. Front Pediatr (2021) 9:799693. doi: 10.3389/fped.2021.799693 35071140 PMC8777267

[16] Floros J Thorenoor N Tsotakos N Phelps DS. Human Surfactant Protein SP-A1 and SP-A2 Variants Differentially Affect the Alveolar Microenvironment, Surfactant Structure, Regulation and Function of the Alveolar Macrophage, and Animal and Human Survival Under Various Conditions. Front Immunol (2021) 12:681639. doi: 10.3389/fimmu.2021.681639 34484180 PMC8415824

[17] Hoover RR Floros J. Organization of the Human SP-A and SP-D Loci at 10q22-Q23. Physical and Radiation Hybrid Mapping Reveal Gene Order and Orientation. Am J Respir Cell Mol Biol (1998) 18(3):353–62. doi: 10.1165/ajrcmb.18.3.3035 9490653

[18] Floros J Hoover RR. Genetics of the Hydrophilic Surfactant Proteins A and D. Biochim Biophys Acta (1998) 1408(2-3):312–22. doi: 10.1016/s0925-4439(98)00077-5 9813381

[19] DiAngelo S Lin Z Wang G Phillips S Ramet M Luo J. Novel, non-Radioactive, Simple and Multiplex PCR-cRFLP Methods for Genotyping Human SP-A and SP-D Marker Alleles. Dis Markers (1999) 15(4):269–81. doi: 10.1155/1999/961430 PMC3851098 10689550

[20] Floros J Wang G Lin Z. Genetic Diversity of Human SP-A, a Molecule With Innate Host Defense and Surfactant-Related Functions; Characteristics, Primary Function, and Significance. Curr Pharmacogenom (2005) 3(2):87–95. doi: 10.2174/1570160054022935

[21] Floros J Wang G Mikerov AN. Genetic Complexity of the Human Innate Host Defense Molecules, Surfactant Protein A1 (SP-A1) and SP-A2–Impact on Function. Crit Rev Eukaryot Gene Expr (2009) 19(2):125–37. doi: 10.1615/critreveukargeneexpr.v19.i2.30 PMC2967201 19392648

[22] Floros J Phelps D Biebuyck J Lynch C Maze M Saidamn L. Anesthesia: Biologic Foundations. Lippincott-Raven, New York (1997).

[23] Lin Z Pearson C Chinchilli V Pietschmann SM Luo J Pison U. Polymorphisms of Human SP-A, SP-B, and SP-D Genes: Association of SP-B Thr131Ile With ARDS. Clin Genet (2000) 58(3):181–91. doi: 10.1034/j.1399-0004.2000.580305.x 11076040

[24] Lahti M Marttila R Hallman M. Surfactant Protein C Gene Variation in the Finnish Population - Association With Perinatal Respiratory Disease. Eur J Hum Genet (2004) 12(4):312–20. doi: 10.1038/sj.ejhg.5201137 14735158

[25] Puthothu B Krueger M Heinze J Forster J Heinzmann A. Haplotypes of Surfactant Protein C are Associated With Common Paediatric Lung Diseases. Pediatr Allergy Immunol (2006) 17(8):572–7. doi: 10.1111/j.1399-3038.2006.00467.x 17121584

[26] Silveyra P Floros J. Genetic Variant Associations of Human SP-A and SP-D With Acute and Chronic Lung Injury. Front Biosci (Landm Ed) (2012) 17:407–29. doi: 10.2741/3935 PMC3635489 22201752

[27] Lahti M Lofgren J Marttila R Renko M Klaavuniemi T Haataja R. Surfactant Protein D Gene Polymorphism Associated With Severe Respiratory Syncytial Virus Infection. Pediatr Res (2002) 51(6):696–9. doi: 10.1203/00006450-200206000-00006 12032263

[28] Thomas NJ DiAngelo S Hess JC Fan R Ball MW Geskey JM. Transmission of Surfactant Protein Variants and Haplotypes in Children Hospitalized With Respiratory Syncytial Virus. Pediatr Res (2009) 66(1):70–3. doi: 10.1203/PDR.0b013e3181a1d768 PMC2710771 19287351

[29] El Saleeby CM Li R Somes GW Dahmer MK Quasney MW DeVincenzo JP. Surfactant Protein A2 Polymorphisms and Disease Severity in a Respiratory Syncytial Virus-Infected Population. J Pediatr (2010) 156(3):409–14. doi: 10.1016/j.jpeds.2009.09.043 19914637

[30] Ampuero S Luchsinger V Tapia L Palomino MA Larrañaga CE. SP-A1, SP-A2 and SP-D Gene Polymorphisms in Severe Acute Respiratory Syncytial Infection in Chilean Infants. Infect Genet Evol (2011) 11(6):1368–77. doi: 10.1016/j.meegid.2011.04.033 21601013

[31] Kala P Ten Have T Nielsen H Dunn M Floros J. Association of Pulmonary Surfactant Protein A (SP-A) Gene and Respiratory Distress Syndrome: Interaction With SP-B. Pediatr Res (1998) 43(2):169–77. doi: 10.1203/00006450-199802000-00003 9475280

[32] Nogee LM Wert SE Proffit SA Hull WM Whitsett JA. Allelic Heterogeneity in Hereditary Surfactant Protein B (SP-B) Deficiency. Am J Respir Crit Care Med (2000) 161(3 Pt 1):973–81. doi: 10.1164/ajrccm.161.3.9903153 10712351

[33] Rämet M Haataja R Marttila R Floros J Hallman M. Association Between the Surfactant Protein A (SP-A) Gene Locus and Respiratory-Distress Syndrome in the Finnish Population. Am J Hum Genet (2000) 66(5):1569–79. doi: 10.1086/302906 PMC1378016 10762543

[34] Floros J Fan R Diangelo S Guo X Wert J Luo J. Surfactant Protein (SP) B Associations and Interactions With SP-A in White and Black Subjects With Respiratory Distress Syndrome. Pediatr Int (2001) 43(6):567–76. doi: 10.1046/j.1442-200x.2001.01474.x PMC2907917 11737731

[35] Amatya S Ye M Yang L Gandhi CK Wu R Nagourney B. Single Nucleotide Polymorphisms Interactions of the Surfactant Protein Genes Associated With Respiratory Distress Syndrome Susceptibility in Preterm Infants. Front Pediatr (2021) 9:682160. doi: 10.3389/fped.2021.682160 34671583 PMC8521105

[36] Lin Z Thorenoor N Wu R DiAngelo SL Ye M Thomas NJ. Genetic Association of Pulmonary Surfactant Protein Genes, SFTPA1, SFTPA2, SFTPB, SFTPC, and SFTPD With Cystic Fibrosis. Front Immunol (2018) 9:2256. doi: 10.3389/fimmu.2018.02256 30333828 PMC6175982

[37] Guo X Lin HM Lin Z Montaño M Sansores R Wang G. Surfactant Protein Gene A, B, and D Marker Alleles in Chronic Obstructive Pulmonary Disease of a Mexican Population. Eur Respir J (2001) 18(3):482–90. doi: 10.1183/09031936.01.00043401 11589345

[38] Seifart C Plagens A Brödje D Müller B von Wichert P Floros J. Surfactant Protein B Intron 4 Variation in German Patients With COPD and Acute Respiratory Failure. Dis Markers (2002) 18(3):129–36. doi: 10.1155/2002/194075 PMC3851100 12515908

[39] Gandhi CK Chen C Amatya S Yang L Fu C Zhou S. SNP and Haplotype Interaction Models Reveal Association of Surfactant Protein Gene Polymorphisms With Hypersensitivity Pneumonitis of Mexican Population. Front Med (2021) 7:588404. doi: 10.3389/fmed.2020.588404 PMC7813780 33469544

[40] Abbasi A Chen C Gandhi CK Wu R Pardo A Selman M. Single Nucleotide Polymorphisms (SNP) and SNP-SNP Interactions of the Surfactant Protein Genes Are Associated With Idiopathic Pulmonary Fibrosis in a Mexican Study Group; Comparison With Hypersensitivity Pneumonitis. Front Immunol (2022) 13:842745. doi: 10.3389/fimmu.2022.842745 35720392 PMC9201215

[41] Gandhi CK Chen C Wu R Yang L Thorenoor N Thomas NJ. Association of SNP-SNP Interactions of Surfactant Protein Genes With Pediatric Acute Respiratory Failure. J Clin Med (2020) 9(4):1183. doi: 10.3390/jcm9041183 PMC7231046 32326132

[42] Gandhi CK Thomas NJ Meixia Y Spear D Fu C Zhou S. SNP–SNP Interactions of Surfactant Protein Genes in Persistent Respiratory Morbidity Susceptibility in Previously Healthy Children. Front Genet (2022) 13:815727. doi: 10.3389/fgene.2022.815727 35401703 PMC8989419

[43] Löfgren J Rämet M Renko M Marttila R Hallman M. Association Between Surfactant Protein A Gene Locus and Severe Respiratory Syncytial Virus Infection in Infants. J Infect Dis (2002) 185(3):283–9. doi: 10.1086/338473 11807709

[44] Selman M Lin HM Montaño M Jenkins AL Estrada A Lin Z. Surfactant Protein A and B Genetic Variants Predispose to Idiopathic Pulmonary Fibrosis. Hum Genet (2003) 113(6):542–50. doi: 10.1007/s00439-003-1015-4 13680361

[45] Jiang Y Chen S Wang X Liu M Iacono WG Hewitt JK. Association Analysis and Meta-Analysis of Multi-Allelic Variants for Large-Scale Sequence Data. Genes (2020) 11(5):586. doi: 10.3390/genes11050586 PMC7288273 32466134

[46] Watson A Kronqvist N Spalluto CM Griffiths M Staples KJ Wilkinson T. Novel Expression of a Functional Trimeric Fragment of Human SP-A With Efficacy in Neutralisation of RSV. Immunobiology (2017) 222(2):111–8. doi: 10.1016/j.imbio.2016.10.015 PMC5152705 27793398

[47] Ginalski K. Comparative Modeling for Protein Structure Prediction. Curr Opin Struct Biol (2006) 16(2):172–7. doi: 10.1016/j.sbi.2006.02.003 16510277

[48] Guex N Peitsch MC Schwede T. Automated Comparative Protein Structure Modeling With SWISS-MODEL and Swiss-PdbViewer: A Historical Perspective. Electrophoresis (2009) 30(Suppl 1):S162–173. doi: 10.1002/elps.200900140 19517507

[49] Berman HM Westbrook J Feng Z Gilliland G Bhat TN Weissig H. The Protein Data Bank. Nucleic Acids Res (2000) 28(1):235–42. doi: 10.1093/nar/28.1.235 PMC102472 10592235

[50] Goh BC Wu H Rynkiewicz MJ Schulten K Seaton BA McCormack FX. Elucidation of Lipid Binding Sites on Lung Surfactant Protein A Using X-Ray Crystallography, Mutagenesis, and Molecular Dynamics Simulations. Biochemistry (2016) 55(26):3692–701. doi: 10.1021/acs.biochem.6b00048 PMC5663190 27324153

[51] Bertoni M Kiefer F Biasini M Bordoli L Schwede T. Modeling Protein Quaternary Structure of Homo- and Hetero-Oligomers Beyond Binary Interactions by Homology. Sci Rep (2017) 7(1):10480. doi: 10.1038/s41598-017-09654-8 28874689 PMC5585393

[52] Bienert S Waterhouse A de Beer TA Tauriello G Studer G Bordoli L. The SWISS-MODEL Repository-New Features and Functionality. Nucleic Acids Res (2017) 45(D1):D313–9. doi: 10.1093/nar/gkw1132 PMC5210589 27899672

[53] Waterhouse A Bertoni M Bienert S Studer G Tauriello G Gumienny R. SWISS-MODEL: Homology Modelling of Protein Structures and Complexes. Nucleic Acids Res (2018) 46(W1):W296–303. doi: 10.1093/nar/gky427 PMC6030848 29788355

[54] Studer G Rempfer C Waterhouse AM Gumienny R Haas J Schwede T. QMEANDisCo-Distance Constraints Applied on Model Quality Estimation. Bioinformatics (2020) 36(8):2647. doi: 10.1093/bioinformatics/btaa058 32048708 PMC7178391

[55] Guex N Peitsch MC. SWISS-MODEL and the Swiss-PdbViewer: An Environment for Comparative Protein Modeling. Electrophoresis (1997) 18(15):2714–23. doi: 10.1002/elps.1150181505 9504803

[56] Voss T Melchers K Scheirle G Schäfer KP. Structural Comparison of Recombinant Pulmonary Surfactant Protein SP-A Derived From Two Human Coding Sequences: Implications for the Chain Composition of Natural Human SP-A. Am J Respir Cell Mol Biol (1991) 4(1):88–94. doi: 10.1165/ajrcmb/4.1.88 1986781

[57] Wang G Bates-Kenney SR Tao JQ Phelps DS Floros J. Differences in Biochemical Properties and in Biological Function Between Human SP-A1 and SP-A2 Variants, and the Impact of Ozone-Induced Oxidation. Biochemistry (2004) 43(14):4227–39. doi: 10.1021/bi036023i 15065867

[58] Wang G Myers C Mikerov A Floros J. Effect of Cysteine 85 on Biochemical Properties and Biological Function of Human Surfactant Protein A Variants. Biochemistry (2007) 46(28):8425–35. doi: 10.1021/bi7004569 PMC2531219 17580966

[59] Kaminski GA Friesner RA Tirado-Rives J Jorgensen WL. Evaluation and Reparametrization of the OPLS-AA Force Field for Proteins *via* Comparison With Accurate Quantum Chemical Calculations on Peptides. J Phys Chem B (2001) 105(28):6474–87. doi: 10.1021/jp003919d

[60] Berendsen HJC van der Spoel D van Drunen R. GROMACS: A Message-Passing Parallel Molecular Dynamics Implementation. Comput Phys Commun (1995) 91(1):43–56. doi: 10.1016/0010-4655(95)00042-E

[61] Van Der Spoel D Lindahl E Hess B Groenhof G Mark AE Berendsen HJ. GROMACS: Fast, Flexible, and Free. J Comput Chem (2005) 26(16):1701–18. doi: 10.1002/jcc.20291 16211538

[62] Hess B Kutzner C van der Spoel D Lindahl E. GROMACS 4: Algorithms for Highly Efficient, Load-Balanced, and Scalable Molecular Simulation. J Chem Theory Comput (2008) 4(3):435–47. doi: 10.1021/ct700301q 26620784

[63] Pronk S Páll S Schulz R Larsson P Bjelkmar P Apostolov R. GROMACS 4.5: A High-Throughput and Highly Parallel Open Source Molecular Simulation Toolkit. Bioinformatics (2013) 29(7):845–54. doi: 10.1093/bioinformatics/btt055 PMC3605599 23407358

[64] Jorgensen WL Chandrasekhar J Madura JD Impey RW Klein ML. Comparison of Simple Potential Functions for Simulating Liquid Water. J Chem Phys (1983) 79(2):926–35. doi: 10.1063/1.445869

[65] Mark P Nilsson L. Structure and Dynamics of the TIP3P, SPC, and SPC/E Water Models at 298 K. J Phys Chem A (2001) 105(43):9954–60. doi: 10.1021/jp003020w

[66] Harrach MF Drossel B. Structure and Dynamics of TIP3P, TIP4P, and TIP5P Water Near Smooth and Atomistic Walls of Different Hydroaffinity. J Chem Phys (2014) 140(17):174501. doi: 10.1063/1.4872239 24811640

[67] Martínez L Borin I Skaf M. Metodos de Qui ´ mica Teo ´ rica e Modelagem ´ Molecular; Morgon, N. H., Coutinho, K. Eds. Editora Livraria da Física (2007) (12):413–452.

[68] Parrinello M Rahman A. Polymorphic Transitions in Single Crystals: A New Molecular Dynamics Method. J Appl Phys (1981) 52(12):7182–90. doi: 10.1063/1.328693

[69] Bosko JT Todd BD Sadus RJ. Molecular Simulation of Dendrimers and Their Mixtures Under Shear: Comparison of Isothermal-Isobaric (NpT) and Isothermal-Isochoric (NVT) Ensemble Systems. J Chem Phys (2005) 123(3):34905. doi: 10.1063/1.1946749 16080761

[70] Evans DJ Holian BL. The Nose–Hoover Thermostat. J Chem Phys (1985) 83(8):4069–74. doi: 10.1063/1.449071

[71] Humphrey W Dalke A Schulten K. VMD: Visual Molecular Dynamics. J Mol Graph (1996) 14(1):33–38, 27-38. doi: 10.1016/0263-7855(96)00018-5 8744570

[72] Singh S Singh VK. "Molecular Dynamics Simulation: Methods and Application,". In: Singh DB Tripathi T, editors. Frontiers in Protein Structure, Function, and Dynamics. Singapore: Springer Singapore (2020). p. 213–38.

[73] David CC Jacobs DJ. Principal Component Analysis: A Method for Determining the Essential Dynamics of Proteins. Methods Mol Biol (Clifton N.J.) (2014) 1084:193–226. doi: 10.1007/978-1-62703-658-0_11 PMC4676806 24061923

[74] Ndagi U Mhlongo NN Soliman ME. The Impact of Thr91 Mutation on C-Src Resistance to UM-164: Molecular Dynamics Study Revealed a New Opportunity for Drug Design. Mol Biosyst (2017) 13(6):1157–71. doi: 10.1039/c6mb00848h 28463369

[75] Palaniappan C Ramalingam R. Deciphering the Molecular Effects of Mutations on ATRX Cause ATRX Syndrome: A Molecular Dynamics Study. J Cell Biochem (2017) 118(10):3318–27. doi: 10.1002/jcb.25984 28294389

[76] Gyimesi G Závodszky P Szilágyi A. Calculation of Configurational Entropy Differences From Conformational Ensembles Using Gaussian Mixtures. J Chem Theory Comput (2017) 13(1):29–41. doi: 10.1021/acs.jctc.6b00837 27958758

[77] Floros J Tsotakos N. Differential Regulation of Human Surfactant Protein A Genes, SFTPA1 and SFTPA2, and Their Corresponding Variants. Front Immunol (2021) 12:766719. doi: 10.3389/fimmu.2021.766719 34917085 PMC8669794

[78] Kersteen EA Raines RT. Contribution of Tertiary Amides to the Conformational Stability of Collagen Triple Helices. Biopolymers (2001) 59(1):24–8. doi: 10.1002/1097-0282(200107)59:1<24::AID-BIP1002>3.0.CO;2-N 11343277

[79] Prescott WA Jr. Doloresco F Brown J Paladino JA. Cost Effectiveness of Respiratory Syncytial Virus Prophylaxis: A Critical and Systematic Review. Pharmacoeconomics (2010) 28(4):279–93. doi: 10.2165/11531860-000000000-00000 20131925

[80] Sorensen GL Dahl M Tan Q Bendixen C Holmskov U Husby S. Surfactant Protein-D-Encoding Gene Variant Polymorphisms Are Linked to Respiratory Outcome in Premature Infants. J Pediatr (2014) 165(4):683–9. doi: 10.1016/j.jpeds.2014.05.042 25015576

[81] Delgado C Krötzsch E Jiménez-Alvarez LA Ramírez-Martínez G Márquez-García JE Cruz-Lagunas A. Serum Surfactant Protein D (SP-D) Is a Prognostic Marker of Poor Outcome in Patients With A/H1N1 Virus Infection. Lung (2015) 193(1):25–30. doi: 10.1007/s00408-014-9669-3 25537934 PMC7102134

[82] Tong M Xiong Y Zhu C Xu H Zheng Q Jiang Y. Serum Surfactant Protein D in COVID-19 Is Elevated and Correlated With Disease Severity. BMC Infect Dis (2021) 21(1):737. doi: 10.1186/s12879-021-06447-3 34344306 PMC8329621

[83] Liu W Bentley CM Floros J. Study of Human SP-A, SP-B and SP-D Loci: Allele Frequencies, Linkage Disequilibrium and Heterozygosity in Different Races and Ethnic Groups. BMC Genet (2003) 4:13. doi: 10.1186/1471-2156-4-13 12908879 PMC194203

[84] Herrera-Ramos E López-Rodríguez M Ruíz-Hernández J Horcajada J Borderías L Lerma E. Surfactant Protein A Genetic Variants Associate With Severe Respiratory Insufficiency in Pandemic Influenza A Virus Infection. Crit Care (2014) 18(3):R127. doi: 10.1186/cc13934 24950659 PMC4229788

[85] Floros J Lin H-M García A Salazar MA Guo X DiAngelo S. Surfactant Protein Genetic Marker Alleles Identify a Subgroup of Tuberculosis in a Mexican Population. J Infect Dis (2000) 182(5):1473–8. doi: 10.1086/315866 11023470

[86] Malik S Greenwood CMT Eguale T Kifle A Beyene J Habte A. Variants of the SFTPA1 and SFTPA2 Genes and Susceptibility to Tuberculosis in Ethiopia. Hum Genet (2006) 118(6):752–9. doi: 10.1007/s00439-005-0092-y 16292672

[87] García-Laorden MI Rodríguez de Castro F Solé-Violán J Rajas O Blanquer J Borderías L. Influence of Genetic Variability at the Surfactant Proteins A and D in Community-Acquired Pneumonia: A Prospective, Observational, Genetic Study. Crit Care (2011) 15(1):R57. doi: 10.1186/cc10030 21310059 PMC3221990

[88] Rämet M Löfgren J Alho O-P Hallman M. Surfactant Protein-A Gene Locus Associated With Recurrent Otitis Media. J Pediatr (2001) 138(2):266–8. doi: 10.1067/mpd.2001.110133 11174628

[89] Pettigrew MM Gent JF Zhu Y Triche EW Belanger KD Holford TR. Association of Surfactant Protein A Polymorphisms With Otitis Media in Infants at Risk for Asthma. BMC Med Genet (2006) 7(1):68. doi: 10.1186/1471-2350-7-68 16884531 PMC1557482

[90] Saxena S Madan T Shah A Muralidhar K Sarma PU. Association of Polymorphisms in the Collagen Region of SP-A2 With Increased Levels of Total IgE Antibodies and Eosinophilia in Patients With Allergic Bronchopulmonary Aspergillosis. J Allergy Clin Immunol (2003) 111(5):1001–7. doi: 10.1067/mai.2003.1395 12743564

[91] Maitra M Wang Y Gerard RD Mendelson CR Garcia CK. Surfactant Protein A2 Mutations Associated With Pulmonary Fibrosis Lead to Protein Instability and Endoplasmic Reticulum Stress. J Biol Chem (2010) 285(29):22103–13. doi: 10.1074/jbc.M110.121467 PMC2903395 20466729

[92] Dy ABC Arif MZ Addison KJ Que LG Boitano S Kraft M. Genetic Variation in Surfactant Protein-A2 Delays Resolution of Eosinophilia in Asthma. J Immunol (2019) 203(5):1122–30. doi: 10.4049/jimmunol.1900546 PMC6702058 31350355

[93] Stein RT Sherrill D Morgan WJ Holberg CJ Halonen M Taussig LM. Respiratory Syncytial Virus in Early Life and Risk of Wheeze and Allergy by Age 13 Years. Lancet (1999) 354(9178):541–5. doi: 10.1016/S0140-6736(98)10321-5 10470697

[94] Sigurs N Bjarnason R Sigurbergsson F Kjellman B. Respiratory Syncytial Virus Bronchiolitis in Infancy Is an Important Risk Factor for Asthma and Allergy at Age 7. Am J Respir Crit Care Med (2000) 161(5):1501–7. doi: 10.1164/ajrccm.161.5.9906076 10806145

[95] Midulla F Nenna R Scagnolari C Petrarca L Frassanito A Viscido A. How Respiratory Syncytial Virus Genotypes Influence the Clinical Course in Infants Hospitalized for Bronchiolitis. J Infect Dis (2018) 219(4):526–34. doi: 10.1093/infdis/jiy496 30204889

[96] Human S Hotard AL Rostad CA Lee S McCormick L Larkin EK. A Respiratory Syncytial Virus Attachment Gene Variant Associated With More Severe Disease in Infants Decreases Fusion Protein Expression, Which May Facilitate Immune Evasion. J Virol (2020) 95(2):e01201–01220. doi: 10.1128/JVI.01201-20 PMC7944440 33115881

[97] Zhang Y Sakthivel SK Bramley A Jain S Haynes A Chappell JD. Serology Enhances Molecular Diagnosis of Respiratory Virus Infections Other Than Influenza in Children and Adults Hospitalized With Community-Acquired Pneumonia. J Clin Microbiol (2017) 55(1):79–89. doi: 10.1128/jcm.01701-16 27795341 PMC5228265

