# Supplementary material for: Hydrophilic But Not Hydrophobic Surfactant Protein Genetic Variants Are Associated With Severe Acute Respiratory Syncytial Virus Infection in Children
Source: Front Immunol. 2022 Jul 12;13:922956. doi: 10.3389/fimmu.2022.922956 (PMC9317530; doi:10.3389/fimmu.2022.922956)
Supplement: Supplementary file 1 [file DataSheet_1.docx]

**Supplementary Table 1:** **PCR primers**

| **Gene** | **Variation ID** | **Nucleotide** | **Primer sequence 5' - 3'** | |
| --- | --- | --- | --- | --- |
| *SFTPA1* | rs1059047 | T/C | F | TCT GCA GGG CTC CAT ATT Gc |
|  |  |  | R | CAC ACA CTG CTC TTT TCC tC |
|  | rs1136450 | C/G | F | GCT GTG CCC TCT GGC CCT Ta |
|  |  |  | R | TCC TTT GAC ACC ATC TC |
|  | rs1136451 | A/G | F | AGA GCG TGG AGA GAA GGG GcA |
|  |  |  | R | GGG TTT GTC TGA TCC CCA TC |
|  | rs1059057 | A/G | F | CAT AAT GAC AGT AGG AGA GAA GGT CTT CTC |
|  |  |  | R | ACC CTC AGT CAG GCC TAC AT |
|  | rs4253527 | C/T | F | GGA GCC TGC AGG TCG GGG AAA Atc G |
|  |  |  | R | TCA GAA CTC ACA GAT GGT CA |
| *SFTPA2* | rs1059046 | C/A | F | ACC TCA TCT TGA TGT CAG CCT CTG GTG CaG |
|  |  |  | R | AGG GCC CAG GTC TCC TCT GA |
|  | rs17886395 | C/G | F | ACC TCA TCT TGA TGT CAG CCT CTG GTG CaG |
|  |  |  | R | AGG GCC AGG GTC TCC TcT GA |
|  | rs1965707 | C/T | F | TTT TCT CTG CAG GCC CCA TGG GTg C |
|  |  |  | R | GGG TTT GTC TGA TCC CCA TC |
|  | rs1965708 | C/A | F | TCT GCA GGG CTC CAT ATT Gc |
|  |  |  | R | CAC ACA CTG CTC TTT TCC tC |
| *SFTPB* | rs2077079 | A/C | F | GTC CAG CTA TAA GGG GCC GTG |
|  |  |  | R | GTG AGT GGT GGA GCT GCC TA |
|  | rs3024798 | C/A | F | ACT CTT GTG TCC TCC ACC TTG |
|  |  |  | R | GGC ATA GGT CAT CCT GGG CA |
|  | rs1130866 | C/T | F | CTC GAA TTC ACT CGT GAA CTC CAG CAC CC |
|  |  |  | R | GTG AGC TTG CAG CCC TCT CA |
| *SFTPC* | rs4715 | C/A | F | GCT GAT CGC CTA CAA GCC CAG |
|  |  |  | R | CTG GAA GTT GTG GAC TTT aCT A |
|  | rs1124 | G/A | F | GAT GGA ATG CTC TCT GCA GG |
|  |  |  | R | GCA CCT CGC CAC ACA GGG aG |
| *SFTPD* | rs721917 | T/C | F | CTC CTC TCT GCA CTG GTC AT |
|  |  |  | R | ACC AGG GTG CAA GCA CTG cG |
|  | rs2243639 | A/G | F | AGC GTG GAG TCC CTG GAA gC |
|  |  |  | R | AGA TTC TCT CCA TGT TCC CAG |

Lower case: mismatch to DNA sequence

**Supplementary Table 2:** **Primers for the *SFTPA1, SFTPA2, SFTPB, SFTPC* and *SFTPD* genes**

| **Target ID** | **Upstream Locus-Specific Oligo** | **Downstream Locus-Specific Oligo** |
| --- | --- | --- |
| SFTPB_1.98494 | GGGAGGTGGAGTGAGTGCTGTTCTT | CATCAGGAAAACACCCTTTCCGGAC |
| SFTPB_2.131669 | GAGCCCTAGAGTGATCCAGAGATGT | AAAATCATGAGAGCATGGAAAAGGG |
| SFTPB_3.191567 | CAGAAACCAAACACCTCTGTAGGGT | AGACAATGGTGGGACTGTAAGTTAG |
| SFTPB_4.150372 | GAGGCTTGCCAAGTGAAGGTCCCAT | GACAGAAACCAAACACCTCTGTAGG |
| SFTPB_5.11350 | GAGCCGGGAAGGTGATAGGAAGCTC | GAGAGAGCCGCTTGTGCATTGCCCC |
| SFTPB_6.163546 | TGCCCCAGGACTCAAACGGCATCTG | GGGAAGGTGATAGGAAGCTCCAGGC |
| SFTPB_6.138664 | CACATAGAGTTCTAAACCACAGCAC | TAAAGTGGAGATCCAGAGGGCTAGA |
| SFTPB_7.217432 | GATTAGGGGTCAGTCTGCCCTGGTG | AAGCCGGCCAGACACAAACACACAG |
| SFTPB_8.113325 | CCTCTTCCTAAGAGAACCTCCCCAT | GCTCAATAGCACAGCCAGAGATGGT |
| SFTPB_9.246239 | GTGAAATCATGATGCCAGGTGTGTA | CATAGGGGATGGATGAGATGAAATG |
| SFTPB_10.60561 | AGCAAAGACAAACACTGAGGTCGCT | GGCATTAACCCAAGTCTGTCCTCAT |
| SFTPC_1.23508 | TGAAGTTTCTCATGGCTTCTGACTC | ACCAGGGTCTTATATGTGTCCGTAG |
| SFTPC_2.226047 | ACTCCCCTTGGACAGTTTCCTATCG | CCCTATACAAGCTGAACACCCTCCC |
| SFTPC_3.6574 | GGGCACGGGAGTCATACTAGAATCT | GTACTCAAATGCGCTTCCCTCTTTC |
| SFTPC_4.239595 | AATGAGGAACAGTGCTTTACAGGTG | AGTCATACTAGAATCTGGGGCTCAG |
| SFTPC_5.304747 | ACACCTGCTCACCGGAGGCGTCCTA | TAGTGAGTGGACTCAGCTGAGCCCA |
| SFTPC_5.300193 | CTGTGAAGCAAAGGAATGTTGAGGA | TAGTAGAGCGGCACCTCGCCACACA |
| SFTPA2_1.468276 | TCCGCTACTCAGATGGGACCCCTGT | CAAACCATCCCAGCACTGCACCCCA |
| SFTPA2_1.735711 | GGTAGCAGAGACCCTAGGTGAGGGA | TGCAGGTCGGGGAAAAGAGCAGTGT |
| SFTPA2_2.197727 | GACACACTGGAATCTCGTGGACCCT | GAATAGAGATGACAAACAAGCGTGC |
| SFTPA2_3.434047 | CTCATAGTGCCCACGGAGTGATAGC | ATTTGGCTCAACCTAAGTAAGAGAA |
| SFTPA2_4.298790 | CCCTCTGGCCCTCACCCTCATCTTG | GGAGTGATAGCTGAGCCAGCCCCGG |
| SFTPA2_4.348137 | CATGCCAGGTGCCAGGTGATGCTTG | AGAGATGGTGTCAAAGGAGACCCTG |
| SFTPA1_1.311545 | TCGCTGCTCCTGCAGGAGGGATGGC | AATCTTCCGTGCTCTGCAGAAAACC |
| SFTPA1_1.204626 | CAGGGTCTCCTTTGAGACCATCTCT | ATTCCCAGCATCACCTGGCACCTGG |
| SFTPA1_2.18126 | CCAGGGCTGGCTCAGCTATCACTCT | AGGCTGCCATCAAGATGAGGTTGAG |
| SFTPA1_3.151334 | TAGGGTTTGTCTGATCCCCATCATC | GCTATCACTCTATCACTCCGTGGGC |
| SFTPA1_4.125495 | GGGAGTGAATTGTGTCTGCTTCTGT | GTGTTTTGCCATCTGCAATCCTGGA |
| SFTPA1_5.52148 | GACCTGCGGGCTCCCCTCGGTACCA | TGGGGTCTCTGCTACCAGATTCTCC |
| SFTPA1_5.552915 | GGGAAGAGTCAGGGCCCATCAGAGG | GTCTGAGTAGCGGAAGTCTCCAGGG |
| SFTPD_1.80565 | CCAAGACAGAGGGCAAGTTCACCTA | CTCTCCAGAATGTGGCCAAAAGTTA |
| SFTPD_1.412117 | GAAGATTTTCAAGACAGCAGGCTTT | CTTGTGGTCTGCGAGTTCTGAGCCA |
| SFTPD_1.725192 | GGCTGTTCTGGAACAATCTCTCTTG | GAGCATGACTGATTCCAAGACAGAG |
| SFTPD_2.312002 | GTGAAGTACAGGCAGCAATTTGTTC | CACTAGATCTTGACCTTGGTTTGGC |
| SFTPD_3.104735 | CATCTGTCTATTTCTGGGCAATAAG | CCCATTAATGGCAGCTGGTGGGTGG |
| SFTPD_4.161939 | AAAGCTCCATTGCTAACAAAACCAC | CTCTGGGGACTGTTCCTATCTAGGG |
| SFTPD_5.307455 | CTTACCTCTGTTATTACCCTCACCT | CACAACTGCAATATTTTCCTCTCAC |
| SFTPD_6.123444 | CTGGGTGTGTGACGGTGGAAGTCTC | CAGGAAGAAGGTTGTCTGAAGGTGG |
| SFTPD_7.289157 | CTAAACCATGTCCATGAAGCATAAG | TATGGAAGTCTGGAGACGTGTTTCT |

**Supplementary Table 3: Allele frequencies in children with moderate and severe RSV**

|  | **Children with Moderate RSV** | | | | **Children with Severe RSV** | | | |
| --- | --- | --- | --- | --- | --- | --- | --- | --- |
| **SNP ID** | **Ref** | **Ref Freq** | **Alt** | **Alt Freq** | **Ref** | **Ref Freq** | **Alt** | **Alt Freq** |
| rs1130866 | C | 0.5203 | T | 0.4797 | C | 0.5333 | T | 0.4667 |
| rs3024798 | A | 0.6351 | C | 0.3649 | A | 0.7593 | C | 0.2407 |
| rs2077079 | C | 0.5676 | A | 0.4324 | C | 0.6389 | A | 0.3611 |
| rs4715 | A | 0.7533 | C | 0.2467 | A | 0.7472 | C | 0.2528 |
| rs1124 | A | 0.6959 | G | 0.3041 | A | 0.6878 | G | 0.3122 |
| rs1965708 | A | 0.7892 | C | 0.2108 | A | 0.8111 | C | 0.1889 |
| rs1965707 | T | 0.7319 | C | 0.2681 | T | 0.7222 | C | 0.2778 |
| rs17886395 | C | 0.5482 | G | 0.4518 | C | 0.64 | G | 0.36 |
| rs1059046 | C | 0.5659 | A | 0.4341 | C | 0.5819 | A | 0.4181 |
| rs1059047 | C | 0.91667 | T | 0.08333 | C | 0.8929 | T | 0.1071 |
| rs1136450 | C | 0.5365 | G | 0.4635 | C | 0.5625 | G | 0.4375 |
| rs1136451 | G | 0.8304 | A | 0.1696 | G | 0.7928 | A | 0.2072 |
| rs1059057 | G | 0.90361 | A | 0.09639 | G | 0.8973 | A | 0.1027 |
| rs4253527 | T | 0.91765 | C | 0.08235 | T | 0.8851 | C | 0.1149 |
| rs2243639 | A | 0.5842 | G | 0.4158 | A | 0.68 | G | 0.32 |
| rs721917 | C | 0.6029 | T | 0.3971 | C | 0.5498 | T | 0.4502 |

Ref: Reference, Ref Freq: Reference Frequency, Alt: Alternative, Alt Freq: Alternative Frequency

**Supplementary Table 4: Association of genotype with RSV Severity using the multivariate logistic analysis after adjusting for covariates (age, ethnicity, co-infection, smoke and pet exposure)**

| **Gene** | **SNPs** | **Genotype** | **OR (95% CI)** |
| --- | --- | --- | --- |
| *SFTPA1* | rs1059047 | **CC** | **2.55 (0.2-4.9)** |
|  |  | **CT** | **1.28 (0.1-2.5)** |
|  |  | TT | -2.55 (-4.9--0.2) |
|  | rs1136450 | CC | -0.75 (-2.0-0.5) |
|  |  | CG | -0.37 (-1.0-0.3) |
|  |  | GG | 0.75 (-0.5-2.0) |
|  | rs1136451 | GG | -0.29 (-2.5-1.9) |
|  |  | GA | -0.14 (-1.2-0.9) |
|  |  | AA | 0.29 (-1.9-2.5) |
|  | rs1059057 | GG | -0.83 (-3.6-1.9) |
|  |  | GA | -0.42 (-1.8-1.0) |
|  |  | AA | 0.83 (-1.9-3.6) |
|  | rs4253527 | TT | 0.72 (-1.3-2.7) |
|  |  | TC | 0.36 (-0.6-1.4) |
|  |  | CC | -0.72 (-2.7-1.3) |
| *SFTPA2* | rs1059046 | CC | 0.10 (-1.3-1.5) |
|  |  | CA | 0.05 (-0.6-0.7) |
|  |  | AA | -0.10 (-1.5-1.3) |
|  | rs17886395 | **CC** | **-0.94 (-1.5--0.3)** |
|  |  | **CG** | **-0.47 (-0.8--0.2)** |
|  |  | GG | 0.94 (0.3-1.5) |
|  | rs1965707 | TT | 0.82 (-0.6-2.2) |
|  |  | TC | 0.41 (-0.3-1.1) |
|  |  | CC | -0.82 (-2.2-0.6) |
|  | rs1965708 | AA | -1.10 (-2.7-0.5) |
|  |  | AC | -0.55 (-1.3-0.2) |
|  |  | CC | 1.10 (-0.5-2.7) |
| *SFTPB* | rs2077079 | CC | 1.01 (-0.9-2.9) |
|  |  | CA | 0.51 (-0.4-1.5) |
|  |  | AA | -1.01 (-2.9-0.9) |
|  | rs3024798 | AA | -2.02 (-4.1-0.0) |
|  |  | AC | -1.01 (-2.0-0.0) |
|  |  | CC | 2.02 (0.0-4.1) |
|  | rs1130866 | CC | -0.02 (-0.8-0.7) |
|  |  | CT | -0.01 (-0.4-0.4) |
|  |  | TT | 0.02 (-0.7-0.8) |
| *SFTPC* | rs4715 | AA | 0.07 (-1.2-1.4) |
|  |  | AC | 0.03 (-0.6-0.7) |
|  |  | CC | -0.07 (-1.4-1.2) |
|  | rs1124 | AA | -0.09 (-1.4-1.2) |
|  |  | AG | -0.05 (-0.7-0.6) |
|  |  | GG | 0.09 (-1.2-1.4) |
| *SFTPD* | rs721917 | CC | 0.35 (-0.4-1.1) |
|  |  | CT | 0.18 (-0.2-0.5) |
|  |  | TT | -0.35 (-1.1-0.4) |
|  | rs2243639 | **AA** | **-0.91 (-1.8--0.1)** |
|  |  | **AG** | **-0.46 (-0.9--0.1)** |
|  |  | GG | 0.91 (0.0-1.8) |

SNPs: Single nucleotide polymorphisms, OR: Odds ratio, CI: Confidence interval, Bold text is statistically significant with *p* ≤ 0.05.
