# Supplementary material for: Hydrophilic But Not Hydrophobic Surfactant Protein Genetic Variants Are Associated With Severe Acute Respiratory Syncytial Virus Infection in Children
Source: Front Immunol. 2022 Jul 12;13:922956. doi: 10.3389/fimmu.2022.922956 (PMC9317530; doi:10.3389/fimmu.2022.922956)
Supplement: Supplementary file 2 [file DataSheet_2.docx]

**Supplementary file: The analysis of SP-A simulated structural data**

1. **Root-mean-square deviation (RMSD) of the center of mass of residues**


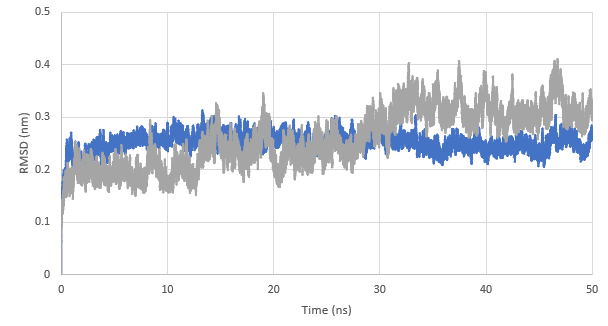

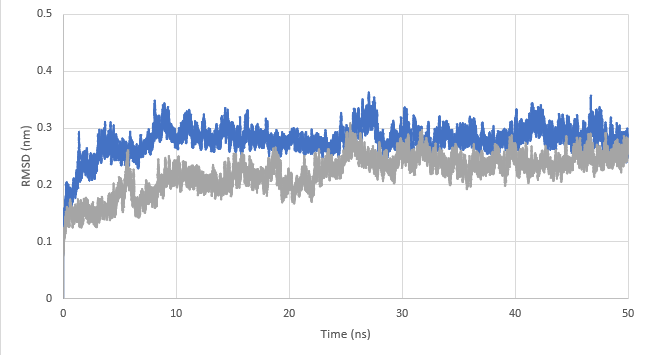


**A**

**B**

**C**


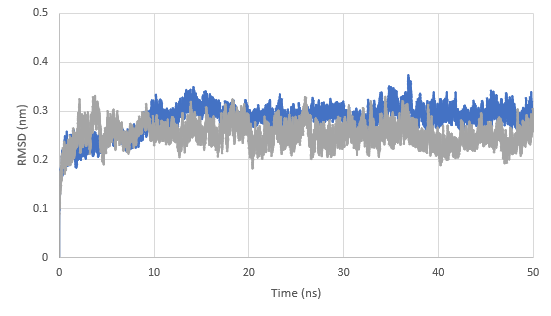


**Figure 1S.** Root-mean-square deviation (RMSD) of the center of mass of residues in monomers during 50 ns of simulation of SP-A trimers. Panels A, B, and C show data from monomers 1, 2, and 3, respectively. Blue and gray colors correspond to Q223 and K223 protein monomers, respectively. The y-axis depicts RMSD values in length units and the x-axis the time lapsed in nanosecond (ns).

RMSD measures fluctuation of the entire targeted structure compared to reference structure over time. Although both proteins oscillate differently, the assumed values did not differ significantly between Q223 and K223 or among the monomers of each variant throughout the simulation. Monomers oscillated close to the 0.25 value, although monomer 2 of the K223 SP-A showed a slightly greater mobility than the others in the middle of the simulation. Since the Q223K variants had no significant impact on RMSD, we conclude that a single change in the specific amino acid (223) does not significantly influence atomic movement of the protein in relation to the center of mass.

**2) Radius of gyration**

**A**

**B**

**C**


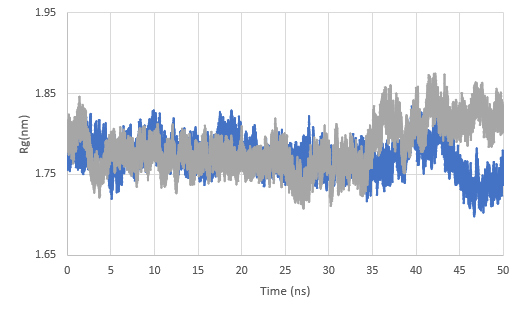

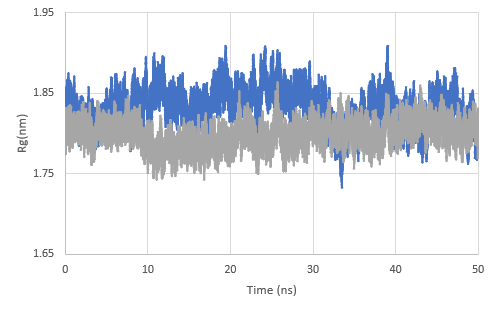

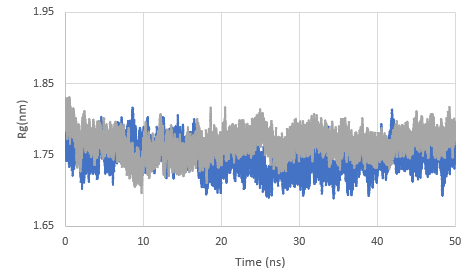


Figure 2S. Radius of gyration (R_g_) of the center of mass of monomers during 50 nanosecond (ns) of simulation of SP-A trimers. Panels A, B, and C depict data for monomers 1, 2, 3, respectively. Blue and gray colors correspond to Q223 and K223 protein monomers, respectively.

The Rg of a protein is the measure of the stability of protein folding. If a protein is stably folded, it will likely maintain a relatively steady value of

**Figure 2S**. Radius of gyration (R_g_) of the center of mass of monomers during 50 nanosecond (ns) of simulation of SP-A trimers. Panels A, B, and C depict data for monomers 1, 2, 3, respectively. Blue and gray colors correspond to Q223 and K223 protein monomers, respectively.

The Rg of a protein is the measure of the stability of protein folding. If a protein is stably folded, it will likely maintain a relatively steady value of Rg. The Rg data show very small variations in the K223 SP-A compared to the Q223 SP-A monomers resulting in similar values and similar patterns. The K223 SP-A monomer 1, however, at the end of the simulation, showed an increase in the values compared to Q223 monomer 1 indicating increased unfolding of K223 SP-A. Furthermore, approximately in the first half of the trimer simulation, the Rg for monomers 1 and 3 is oscillating in the same range for both proteins indicating minimal effect of Q223K on these two monomers but monomer 2 showed a more noticeable change in Rg than the others. In the latter case, the K223 SP-A has slightly lower values, indicating that this monomer is more folded than its Q223 counterpart. However, none of the observed changes is significant indicating that the Q223K change does not cause major changes in the SP-A structural behavior.

**3) Hydrogen bonds**

**A**

**B**

**C**


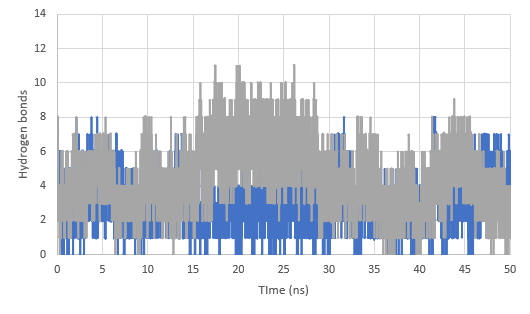

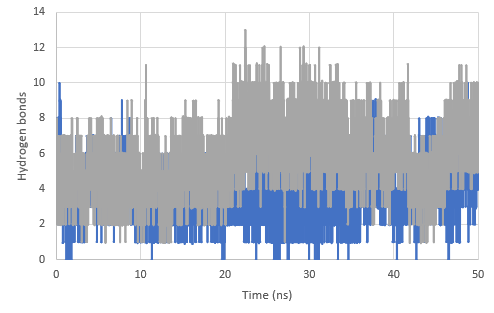

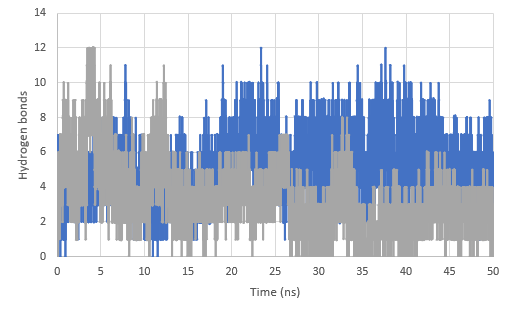


Figure 3S. Analysis of hydrogen bonds as a function of time. The number of hydrogen bonds between monomers 1 and 2 (A), 1 and 3 (B), and 2 and 3 (C) during 50 ns of simulation of SP-A protein trimers. Blue and gray colors correspond to Q223 and K223 protein monomers, respectively. The number on Y-axis indicates the average number of hydrogen bonds present in each timeframe (X-axis) of the trimer simulation.

The highest number of hydrogen bonds formed between monomers were n=13 for the K223 variant (Panel B, marked with a black arrow), and for the Q223 variant n=12 (Panel C, marked with a red arrow). Thus, the amino acid change causes small conformational changes in some regions either an increase in the number of hydrogen bonds present between some monomer pairs (Panels A and B) or a decrease (Panel C) and the pattern of this increase/decrease reverses towards the end of the simulation (Panels A-C). The average number of hydrogen bonds oscillates around 4 to 6 indicating that the majority of hydrogen bonds between monomers are formed and broken during simulation. Although the K223 trimer forms a greater number of hydrogen bonds than the Q223, the difference in the number of hydrogen bonds between Q223 and K223 variants is subtle. However, this may contribute to a higher stability of the K223 macromolecule.

**4) Database of Secondary Structure in Proteins (DSSP)**

Analysis of the secondary structure of each monomer throughout the simulation of each SP-A trimer is shown in Figure 4S (Panels A, B, and C). The top graph in each panel (A-C) depicts the data for each monomer (1-3), respectively, of the Q223 variant, while the bottom graph depicts the corresponding data for the K223 variant. The y-axis shows the amino acid residue number and x-axis the time lapsed in picosecond (ps).

**
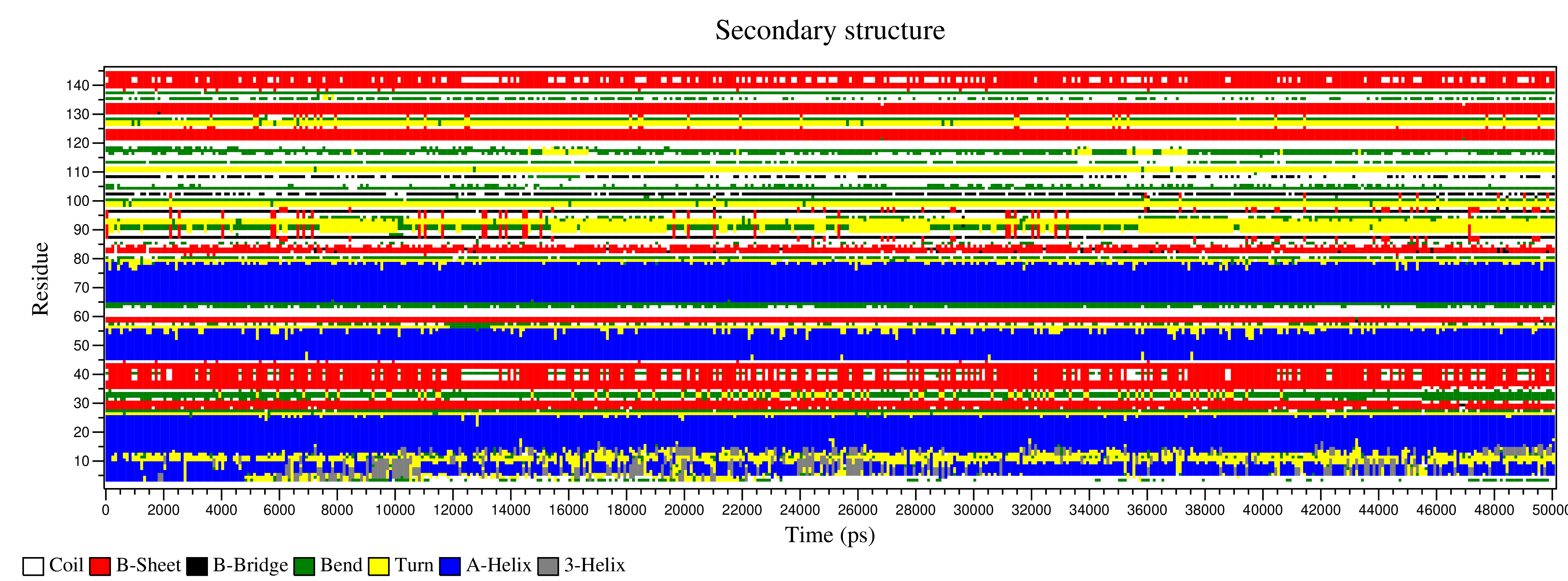

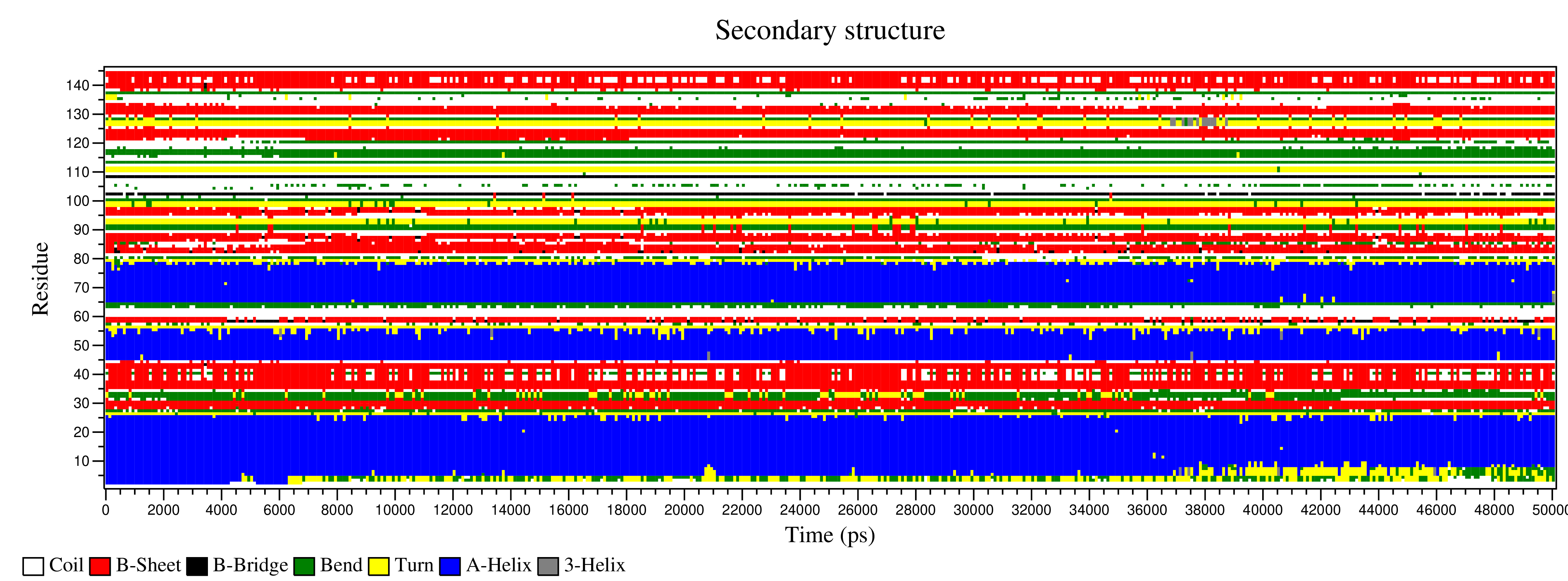
A**

**
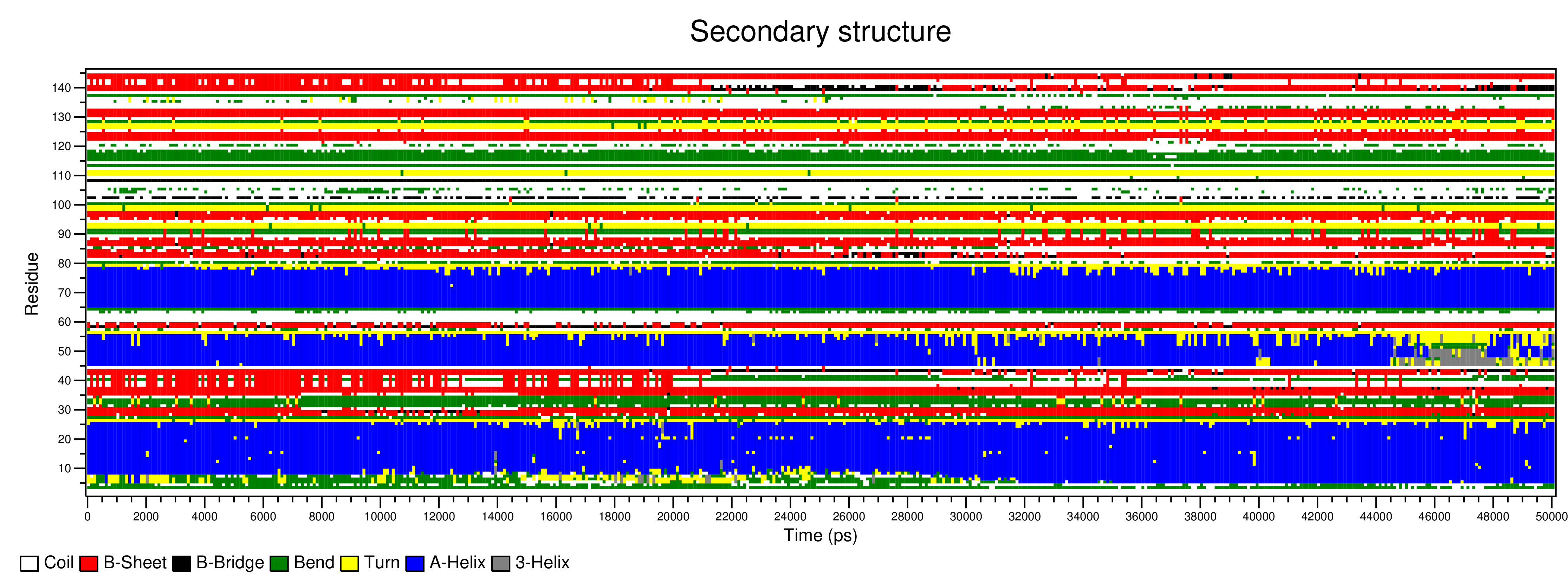

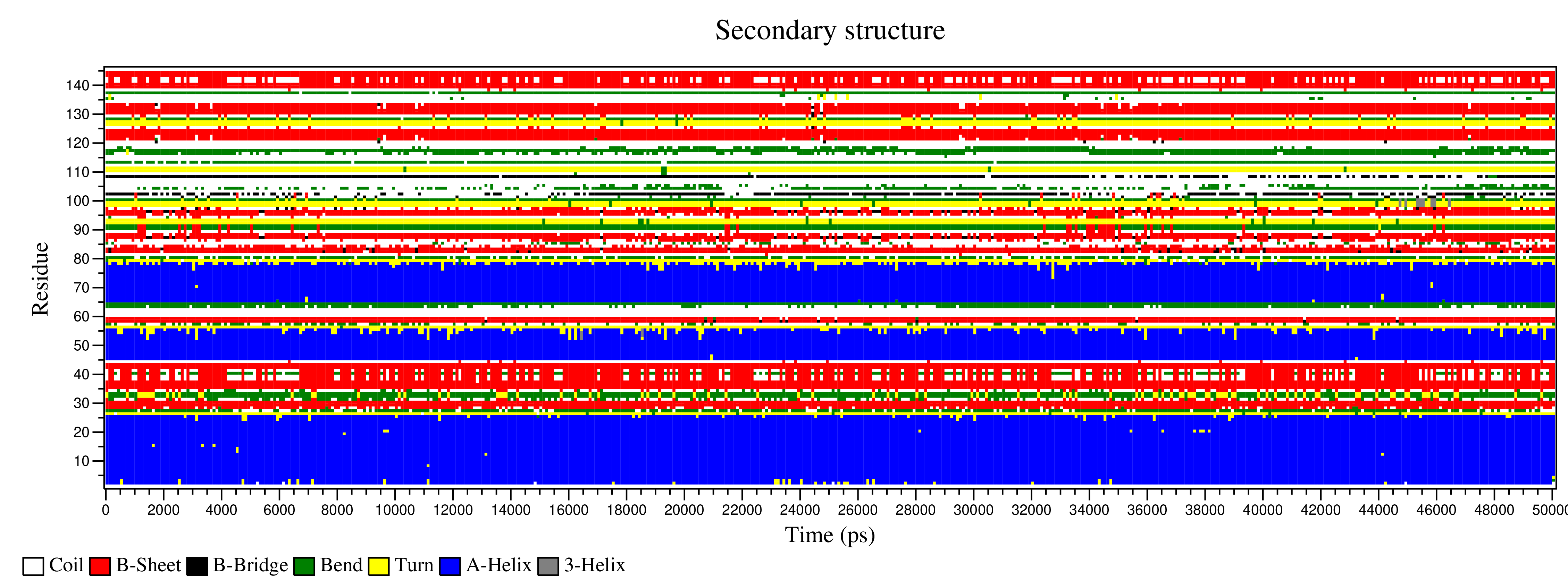
B**

**
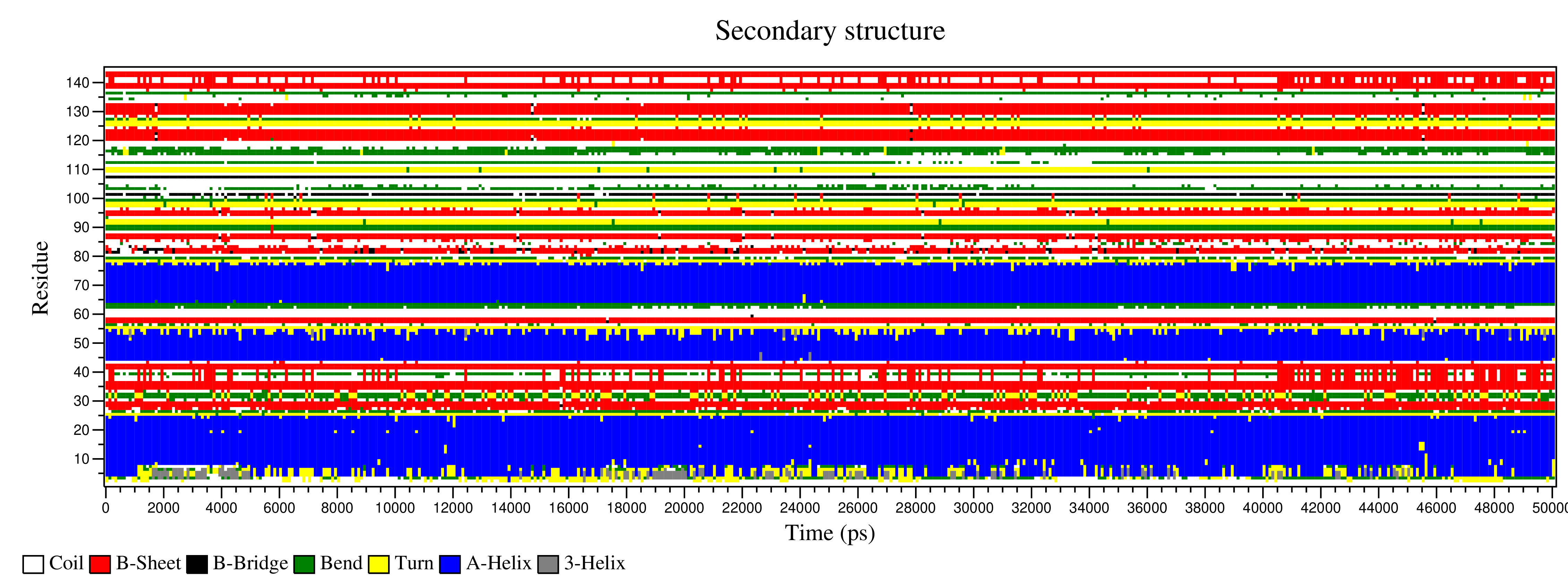

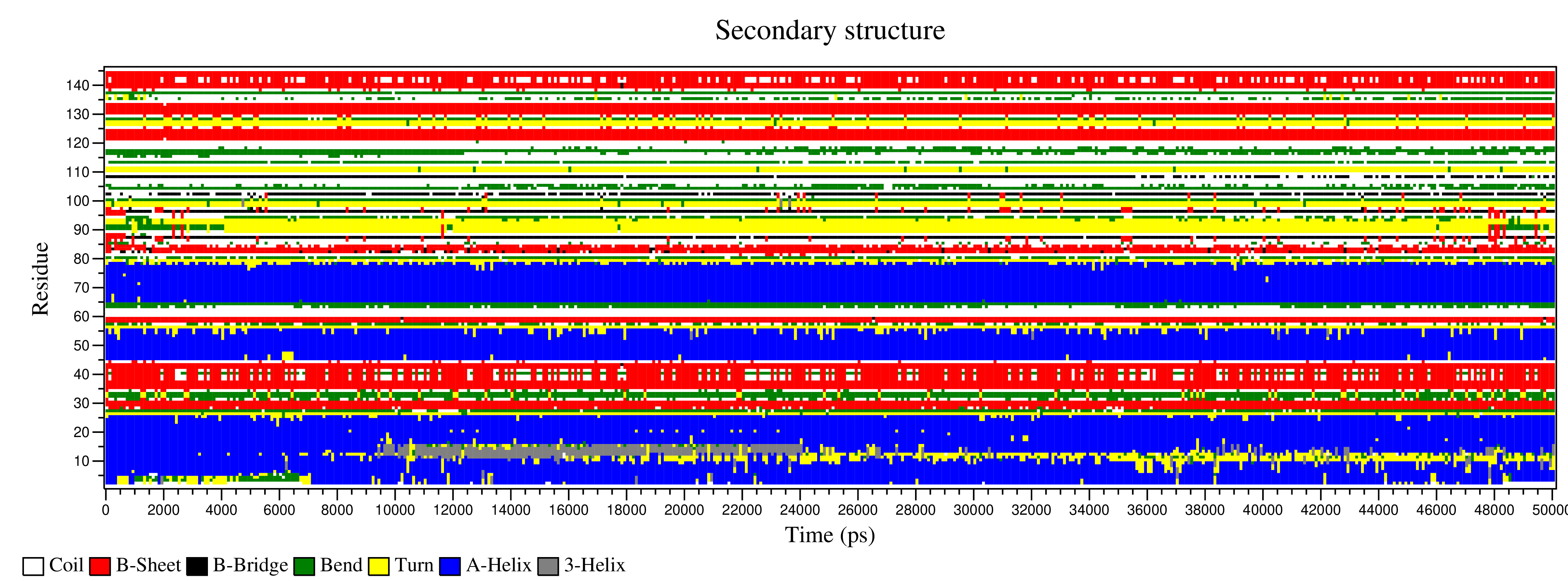
C**

For both Q223 and K223 SP-A, we observed the presence of 3 large bands corresponding to the alpha helix (shown in blue color). The region near the amino acids 0, 30, 40 and 140 that correspond to SP-A residues 104, 134, 144, and 244 from the protein data bank structure, respectively, oscillate between two or three different structures for both Q223 and the K223 SP-A. For example, amino acid 140 changes from coil (shown in white color) to beta sheet (b-sheet, shown in red color) throughout the simulation in both trimer proteins. Amino acids 30 and 40 change among coil (white), b-sheet (red), and bend (shown in green color) conformation structures. Of interest, the “bend” conformation (green color) predominates at amino acid 223 (residue 119 in Panels A-C), the site of genetic variant.

**5) Principal Component Analysis (PCA)**

The principal component (PC) analysis of each monomer throughout the simulation of each SP-A trimer is shown in Figure 5S (Panels A, B, and C). Each panel (A-C) depicts the data for each monomer (1-3), respectively, of both proteins (the Q223 variant shown in blue color and the K223 shown in gray color). The top and bottom graphs of each panel represent data for the vector 1 (PC 1) and vector 2 (PC 2), respectively. Vectors are represented on y-axis and x-axis the time lapsed in nanosecond (ns).


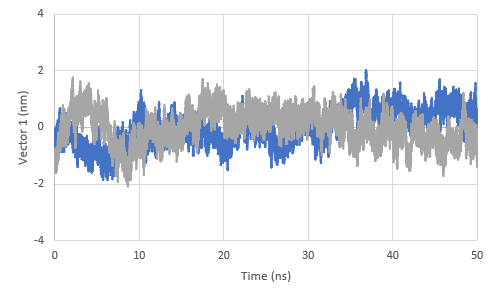

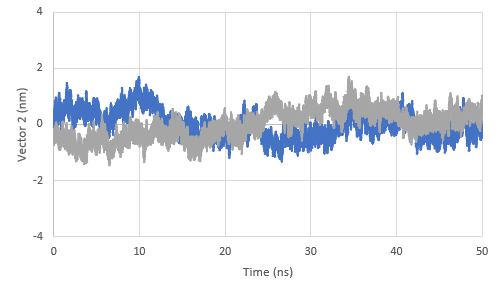

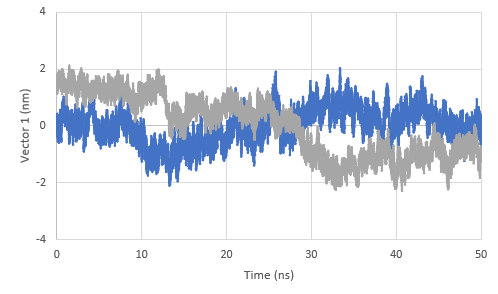

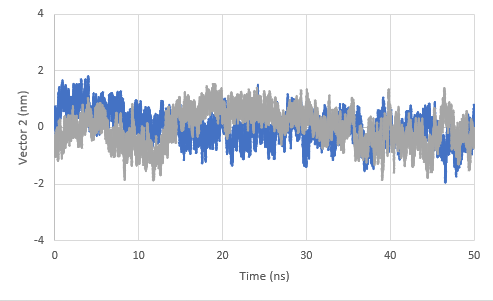


**A**

**B**

**C**


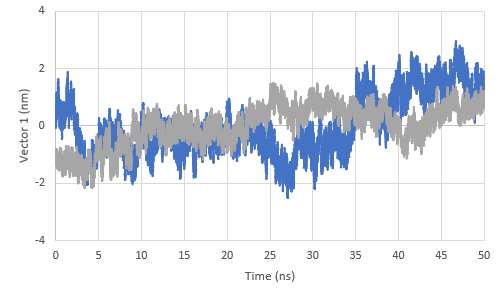

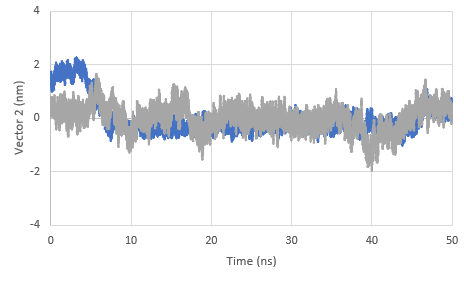


PCA converts a set of correlated observations (movement of all atoms in protein) to a set of principal components which are linearly independent (or uncorrelated). PCA reveals the most important motions in protein during simulation. Mathematically, it is a transformation of the data to a new coordinate system, in which the first coordinate represents the greatest variance, the second coordinate represents the second most variance, and so on. For the current analysis, the vector 1 is a linear combination of the most important residue motions and vector 2 is a linear combination of the second most important residue motions of the SP-A trimer.

We did not observe drastic change in vector values between the Q223 and K223 SP-A indicating that the three monomers behaved similarly and variant did not cause major changes in SP-A behavior.
